# Supplementary material for: Predicted impact of banning nonessential, energy-dense food and beverages in schools in Mexico: A microsimulation study
Source: PLoS Med. 2024 May 10;21(5):e1004394. doi: 10.1371/journal.pmed.1004394 (PMC11086919; doi:10.1371/journal.pmed.1004394)
Supplement: S1 Appendix — (PDF) [file pmed.1004394.s001.pdf]

S1 Appendix for "Predicted impact of banning processed food and beverages in schools in Mexico: a microsimulation study"

Ana Basto-Abreu, PhD[1,], Martha Carnalla, PhD[1], Francisco Reyes-Sánchez, BS[1,], Alan Reyes-García, MS[1], Michelle M. Haby, PhD[2], Isabel Junquera-Badilla, MSc[1], Lianca Sartoris-Ayala, [1], Juan A. Rivera, PhD[1], Barry M. Popkin, PhD[3], and Tonatiuh Barrientos-Gutiérrez, PhD *[1]

[1] *National Institute of Public Health, Population Health Research Center, Mexico*
[2] *Division of Biological and Health Sciences, Department of Chemical and Biological Sciences, University of Sonora, Hermosillo, Mexico*
[3] *Department of Nutrition, Gillings School of Global Public Health, and the Carolina Population Center, The University of North Carolina at Chapel Hill, Chapel Hill, NC, USA*

*Contact information: Center for Population Health Research, National Institute of Public Health, Avenida Universidad 655, Santa María Ahuacatitlán, 62100 Cuernavaca, Morelos, México, [tbarrientos@insp.mx].

Contents

1 Nutritional criteria to determine banned foods and beverages in schools **3**
1.1 2014 guidelines . . . 3
1.2 2023 guidelines . . . 3

2 Data sources **4**
2.1 Anthropometric data . . . 4

3 Total energy intake by day and calibration **8**
3.1 Energy balance of reference . . . 8
3.2 Energy expenditure of reference . . . 9
3.2.1 Reference fat-free mass and fat mass . . . 10

4 Intervention scenarios **12**
4.1 Using Mexican data to inform the intervention effect . . . 12
4.2 Using Mexican data to the inform intervention effect, varying compliance . . . 12
4.3 Using meta-analytic data to inform the intervention effect . . . 13

5 Microsimulation model **13**
5.1 Summary of Hall et al. equations . . . 14

6 Model validation **14**

7 Baseline vs Business as usual characteristics **16**

8 Uncertainty intervals **16**

9 Data available online **17**

References **18**

1 Nutritional criteria to determine banned foods and beverages in schools

1.1 2014 guidelines

Nutritional criteria considered to determine foods and beverages prohibited within Mexican schools published in "the general guidelines for the sale and distribution of prepared and processed foods and beverages in schools of the National Educational System" in 2014.

Table A: Nutritional criteria established in the 2014 guidelines* to determine foods and beverages not permitted for consumption in schools [1].§

Type of Product	Serving size	Energy	Added sugars	Total Fats	Added trans fats	Added Sodium
Beverages • (per serving size)	250 ml	10 kcal	100 mg from non-caloric sweeteners	–	–	55 mg
Milk (per serving size)	250 ml	125 kcal		4g		
Yogurth (per serving size)	150g: solid<br>200 ml: liquid	80 kcal: solid<br>100 kcal: liquid	30 %kcal	3.75 g: solid<br>2.8 g: liquid		
Nectars (per serving size)	125 ml	70 kcal				
Soy-based drinks (per serving size)	125 ml	100 kcal		3.12g		110 mg
Soy-based drinks with juice (per serving size)	125 ml	60 kcal				62.5 mg
Salty snacks (per serving size)	130 kcal	130 kcal	10 %kcal	35 %kcal<br>15 %kcal from saturated fats	0.5 g	180 mg
Cookies, pastries, candies and desserts (per serving size)	130 kcal	130 kcal	20% kcal	35 %kcal<br>15 %kcal from saturated fats	0.5 g	180 mg
Seeds ‡ (per serving size)	130 kcal	130 kcal	10% kcal	15 %kcal from saturated fats	0.5 g	180 mg
Snack cheese (per serving size)	30 g	80 kcal		68 %kcal<br>39 %kcal from saturated fats		180 mg

*General guidelines for the sale and distribution of prepared and processed foods and beverages in schools of the National Educational System issued in 2014.
• Only secondary and high school are considered for the consumption of sugar-sweetened beverages. In elementary school, the exclusive consumption of water was promoted and the promotion of the consumption of other types of drinks was avoided.
§ Processed foods that met the nutritional criteria could be consumed once a week (Friday).
‡ Only if it contain added -fat, -salt or -sugars.

1.2 2023 guidelines

Nutritional criteria that will be considered in the 2023 reform of the General Education Law, based on the Official Mexican Standard (NOM-051-SCFI/SSA1-2010), "General labeling specifications for prepackaged foods

and non-alcoholic beverages - Commercial information and Sanitation), of 2010 to determine foods and drinks that will be prohibited in schools.

Table B: Nutritional criteria from 2023 reform of the General Education Law to determine foods and beverages that will be banned in schools, based on warning label criteria [2].

Type of product	Energy	Added sugars*	Added saturated fats*	Added trans fats*	Added sodium*
Solid foods (per 100 g)	$\geq$ **275** total kcal	$\geq$ **10% kcal** from free sugars‡	$\geq$ **10% kcal** from saturated fats	$\geq$ **1% kcal** from trans fats	$\geq$ **1 mg** per 1 kcal or $\geq$ **300 mg**<br>Non-caloric beverages: $\geq$ **45 mg**
Beverages (per 100 g)	$\geq$ **70** total kcal<br>$\geq$ **8** kcal from free sugars‡				

*Applied to prepackaged food or beverage with added specified nutrient, those products to which the specified nutrient has been added during the manufacturing process, and added ingredients that contain the specified nutrient.

‡ Monosaccharides and disaccharides added to foods and non-alcoholic beverages by the manufacturer.

2 Data sources

We used anthropometric data of individuals aged 6-18 years old from the Mexican Health and Nutrition Survey (ENSANUT, from its Spanish acronym) 2018. Individuals aged 6-17 were taken as the baseline sample (initial sample) for simulation. Individuals aged 18 years old were considered only to obtain medians of fat mass and fat-free mass (Table F) needed to calibrate the model (calibration is presented in section 3). More details about the anthropometric data are presented in the next subsection.

2.1 Anthropometric data

Trained personnel obtained weight and height from 10,659 individuals between 6 to 18 years old, using standard procedures. [3] To define valid data, anthropometric measurements were transformed to Z scores using the 2006 World Health Organization (WHO) reference standard. [4, 5] We excluded 203 individuals with missing data or with extreme values for height/age (<-6 and >+6 Z score) and bmi/age (<-5 and >+5 Z score), [4, 5]. According to the ENSANUT 2018 methodology, we also excluded children (6-11 years old) with $BMI < 10$ kg/m^2 and $BMI > 38$ kg/m^2, and adolescents (12-18 years old) with $BMI < 10$ kg/m^2 and $BMI > 58$ kg/m^2 (n = 3) [6]. Adolescents who reported being pregnant and/or lactating were also excluded (n = 41). With the remaining sample (n = 10,412), we estimated median values of fat mass and fat-free mass by bmi category, and calibrated Hall's energy equation as described in section 3. Then, we excluded 18-year-old individuals (n = 658) since they were required only to calibrate. The final sample consisted of 9,754 individuals between 6 to 17 years old. The data processing described above is summarized in Figure A.

Figure A: Data processing

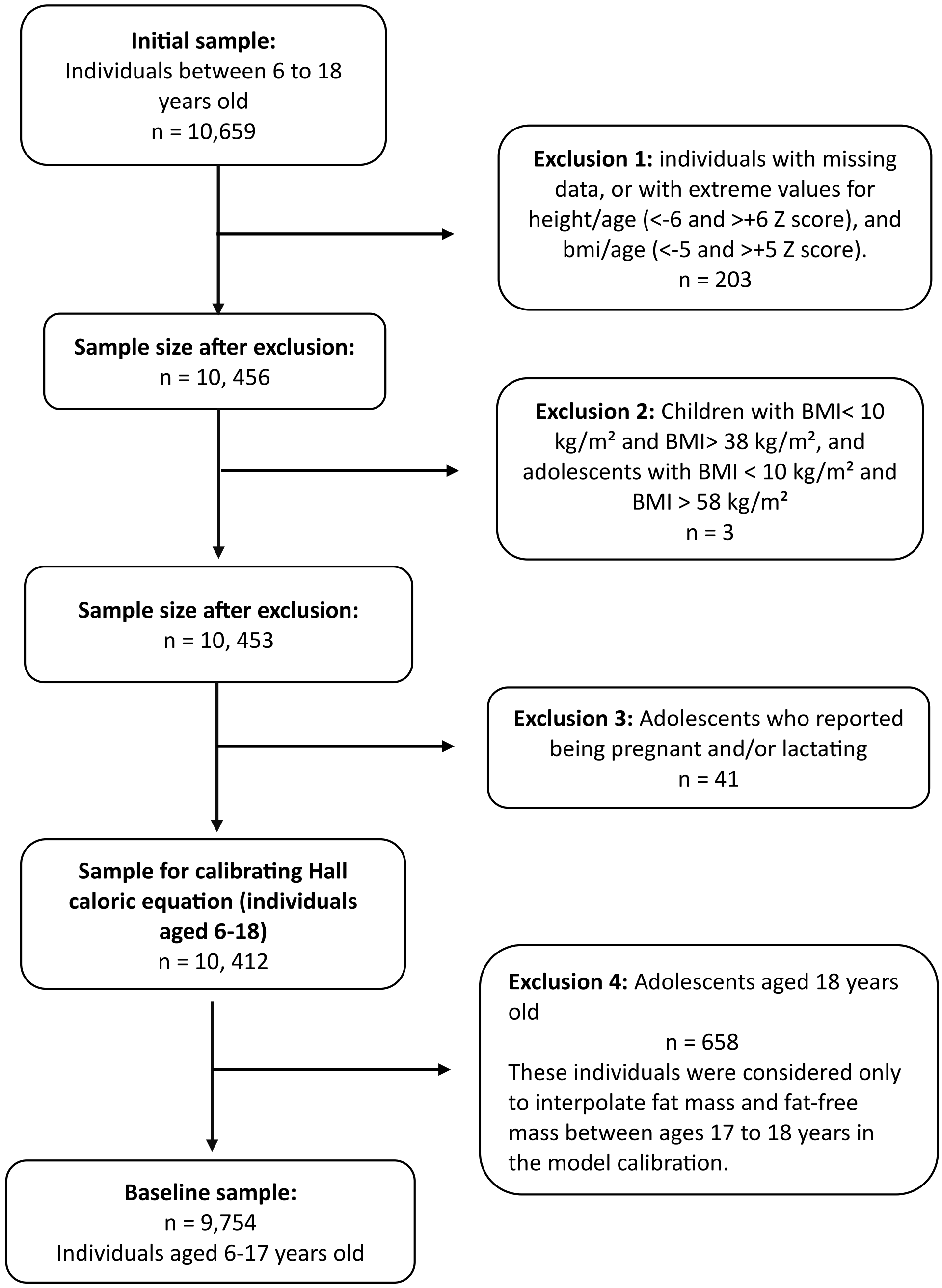

Table C: Model inputs

Input	Value/estimate	Source

**Main scenarios**<br>% TEI from non-essential energy dense food and beverages consumed at schools (with and without compensation)	**Elementary school**<br>1. Foods and beverages<br>-4.2% (95% CI: -4.7, -3.7)<br><br>2. Only foods[a]<br>-3.2% (95% CI: -3.6, - 2.8)<br><br>3. Only beverages[a]<br>-1.0% (95% CI: -1.2, -0.8)<br><br>**Secondary school**<br>1. Foods and beverages<br>-5.4% (95% CI: -6.6, -4.2)<br><br>2. Only foods[a]<br>-3.8% (95% CI: -4.8, -2.8)<br><br>3. Only beverages[a]<br>-1.6% (95% CI: -2.0, -1.2)<br><br>**High school**<br>1. Foods and beverages<br>-4.1% (95% CI: -5.1, -3.1)<br><br>2. Only foods[a]<br>-3.2% (95% CI: -3.2, - 2.0)<br><br>3. Only beverages[a]<br>-1.4% (95% CI: -2.0, -0.8)	ENSANUT 2012 [7]
Compensation (scenario with compensation)	*Foods*<br>a) Change in regulated foods (kcal/capita/day):<br><br>-33.1 (95%CI: -37.7, -28.6)<br><br>b) Change in non-regulated foods :<br>26.8 (95%CI: 20.6, 32.9)<br><br>*Beverages*<br>c) Change in regulated beverages (kcal/capita/day):<br><br>-16.3 (95%CI: -18.7, -13.9)<br><br>d) Change in non-regulated beverages (kcal/capita/day):<br><br>6.3 (95%CI: 4.1, 8.5)	Taillie, L. S. et al. [8]
Measured body weight and height	Mean body weight<br>Mean height	ENSANUT 2018 [9]

**Sensitivity 3. By sex and SES**[b]<br>% TEI from non-essential<br>energy dense food and<br>beverages consumed at<br>schools (with<br>and without compensation)	**Female and Low SES**<br>1. Foods and beverages<br>-4.7% (95% CI: -5.5, -3.9)<br><br>2. Only foods[a]<br>-3.3% (95% CI: -3.9, -2.7)<br><br>3. Only beverages[a]<br>-1.4% (95% CI: -1.8, -1.0)<br><br>**Female and Middle SES**<br>1. Foods and beverages<br>-5.0% (95% CI: -6.1, -3.9)<br><br>2. Only foods[a]<br>-3.7% (95% CI: -4.6, -2.8)<br><br>3. Only beverages[a]<br>-1.3% (95% CI: -1.7, -0.9)<br><br>**Female and High SES**<br>1. Foods and beverages<br>-6.5% (95% CI: -8.0, -5.0)<br><br>2. Only foods[a]<br>-4.7% (95% CI: -5.7, -3.7)<br><br>3. Only beverages[a]<br>-1.9% (95% CI: -2.5, -1.3)<br><br>**Male and Low SES**<br>1. Foods and beverages<br>-2.3% (95% CI: -3.0, -1.6)<br><br>2. Only foods[a]<br>-1.6% (95% CI: -2.1, -1.1)<br><br>3. Only beverages[a]<br>-0.7% (95% CI: -1.2, -0.2)<br><br>**Male and Middle SES**<br>1. Foods and beverages<br>-3.5% (95% CI: -4.4, -2.6)<br><br>2. Only foods[a]<br>-2.6% (95% CI: -3.5, -1.7)<br><br>3. Only beverages[a]<br>-0.8% (95% CI: -1.1, -0.5)<br><br>**Male and High SES**<br>1. Foods and beverages<br>-4.7% (95% CI: -5.7, -3.7)<br><br>2. Only foods[a]<br>-3.4% (95% CI: -4.2, -2.6)<br><br>3. Only beverages[a]<br>-1.3 % (95% CI: -1.7, -0.9)	ENSANUT 2012 [7]

Total energy intake (TEI) from day $t_0 = 0$ (initial time in 2018) to day t_{365}= 365 (one year of simulation)	Average values of TEI (kcal/person) for days 0 (baseline), 1, 2, 100, 200, 300, and 365:<br><br>TEI(0) = 2246<br>TEI (1) = 2246<br>TEI (2) = 2247<br>TEI (100) = 2266<br>TEI (200) = 2286<br>TEI (300) = 2304<br>TEI (365) = 2316	Estimated using a caloric equation proposed by Hall et al. [10]
Compliance rate	At least 30%	Hugues et al. [11]

[a] Used when considering caloric compensation, which was estimated separately for foods and beverages by Tallie et al. [8]

[b] SES = Socioeconomic status

3 Total energy intake by day and calibration

We estimated the daily total energy intake at baseline for each individual in the sample as [10]:

$$I_{ref}(t) = E_{ref}(t) + EB_{ref}(t); \tag{1}$$

where I_{ref} represents the reference energy intake: the required energy intake for children's normal growth, under no intervention. E_{ref} corresponds to the reference energy expenditure, and EB_{ref} denotes the energy balance of reference ($= I_{ref} - E_{ref}$). Equations used for estimating EB_{ref} and E_{ref} are presented in the next subsections. To estimate E_{ref}, average (or median) fat mass and fat-free mass values are needed; we considered median fat mass and fat-free mass by age, sex, and BMI category (calibration). Fat mass and fat-free mass were estimated using the Deurengberg et al. equation [12]. More details are presented in the next subsections.

3.1 Energy balance of reference

EB_{ref} was estimated using an equation adapted from Katan et al.: [10, 13]

$$EB_{ref}(t) = A_{EB} \cdot e^{-(t-t_A^{EB})/\tau_A^{EB}} + B_{EB} \cdot e^{-(t-t_B^{EB})^2/2(\tau_B^{EB})^2} + D_{EB} \cdot e^{-(t-t_D^{EB})^2/2(\tau_D^{EB})^2}. \tag{2}$$

Parameters in Katan's equation are presented in Table D.

Table D: Parameters for the energy balance equation adapted from Katan et al. [13]

Parameter	Males	Females
A_{EB}	7.2	16.5
B_{EB}	30.0	47.0
D_{EB}	21.0	41.0
τ_A^{EB}	15.0	7.0
τ_B^{EB}	1.5	1.0
τ_D^{EB}	2.0	1.5
t_A^{EB}	5.6	4.8
t_B^{EB}	9.8	9.1
t_D^{EB}	15.0	13.5

3.2 Energy expenditure of reference

We estimated the energy expenditure of reference in equation (1) as [10]

$$E_{ref}(t) = K + (\gamma_{FFM} + \delta(t))FFM_{ref}(t) + (\gamma_{FM} + \delta(t))FM_{ref}(t) + \frac{\eta_{FFM}}{\rho_{FFM}}\big(p \cdot EB_{ref}(t) + g(t)\big) + \frac{\eta_{FM}}{\rho_{FM}}\big((1-p)\cdot EB_{ref}(t) - g(t)\big); \quad (3)$$

where K represents an energy expenditure constant dependent on the child's sex (K = 800 kcal/d for males; K = 700 kcal/d for females); γ_F = 4.5 kcal/kg/d and γ_{FFM} = 22.4 kcal/kg/d are regression coefficients explaining the relationship between the resting metabolic rate (dependant variable), and F and FFM, respectively [14]. $FFM_{ref}(t)$ and $FM_{ref}(t)$ are called fat-free mass and fat mass of reference at time t, respectively, and denote the average or median values of FM y FFM by age, sex, and BMI category (see section 3.2.1 for more details). η_{FM}= 180 kcal/kg and η_{FFM}= 230 kcal/kg account for "biochemical efficiencies associated to fat and protein synthesis". ρ_{FM} = 9400 kcal/kg and ρ_{FFM} = $(4.3 \cdot FFM_{ref}(t) + 837)$ kcal/kg are the energy densities for changes in $FFM_{ref}(t)$ and $FM_{ref}(t)$), respectively. p represents the proportion of energy from $EB_{ref}(t)$ going to fat-free mass (energy partitioning ratio), estimated using Forbes equation: [15]

$$p = \frac{C}{C + FM_{ref}(t)}; \quad (4)$$

where

$$C = 10.4 \times \frac{\rho_{FFM}}{\rho_{FM}}.$$

The function for physical activity (δ) in equation (3) is given by:

$$\delta(t) = \delta_{min} + \frac{(\delta_{max} - \delta_{min})P^h}{t^h + P^h}. \quad (5)$$

The minimum physical activity for all ages and sexes is represented by the constant δ_{min} =10 kcal/kg/d. The constant for maximum physical activity is sex specific and given by δ_{max} = 19 kcal/kg/d for males and δ_{max} = 17 kcal/kg/d for females. The parameter P = 12 years represents the point of maximum physical activity whilst the constant h = 10 represents the rate of decline as a function of age.

The term $g(t)$ in equation (3) controls children's body growth, estimated with the equation [10, 13]

$$g(t) = A \cdot e^{-(t-t_A)/\tau_A} + B \cdot e^{-(t-t_B)^2/2\tau_B^2} + D \cdot e^{-(t-t_D)^2/2\tau_D^2}; \quad (6)$$

where the specific parameters for males and females are shown in Table E.

Table E: Parameters for the growth function $g(t)$

Parameter	Males	Females
A	3.2	2.3
B	9.6	8.4
D	10.1	1.1
τ_A	2.5	1.0
τ_B	1.0	0.9
τ_D	1.5	0.7
t_A	4.7	4.5
t_B	12.5	11.7
t_D	15.0	16.2

3.2.1 Reference fat-free mass and fat mass

We estimated daily reference values of fat-free mass (FFM_{ref}) and fat mass ($FM_{ref}(t)$) at a population level, using linear interpolations. First, we estimated median values for fat mass and fat-free mass by age, sex, and BMI category. Fat-free mass and fat mass were estimated for each individual in the sample using Deurengberg et al. equation [12]

$$FM = \begin{cases} \left(\dfrac{1.51 \cdot BMI - 0.7 \cdot age - 2.2}{100}\right) \times BW & \text{if } male \\ \\ \left(\dfrac{1.51 \cdot BMI - 0.7 \cdot age + 1.4}{100}\right) \times BW & \text{if } female \end{cases} \tag{7}$$

$$FFM = BW - FM \tag{8}$$

where BMI and BW stand for body mass index and body weight, respectively. Table F shows the median fat mass and fat-free mass by age, sex and BMI category, obtained with equations (8) and (7). Those median values were estimated using rounded age in years ($= round(age\ in\ months)/12$). The exact age in months was not considered because of the lack of sample in some groups of age, sex, and bmi categories. The median values presented in F were taken as reference values of fat-free mass (FFM_{ref}) and fat mass (FM_{ref}) by age, sex and BMI category (calibration). To get daily values of reference, we used linear interpolation between ages assuming that, under no intervention, the individual would remain in the same body mass category during the simulation period (one year). For example, a 6-year-old boy with underweight at the initial time would have $FFM_{ref}(age = 6) = 14.6$ kg and $FM_{ref}(age = 6) = 2.0$ kg. At age 6.5, these reference values would change to:

$$FFM_{ref}(6.5) = FFM_{ref}(6) + (6.5 - 6.0) \times \big(FFM_{ref}(7.0) - FFM_{ref}(6.0)\big)$$

$$= 14.5 + 0.5 \times (16.4 - 14.5) = 15.4,$$

$$FM_{ref}(6.5) = FM_{ref}(6) + (6.5 - 6.0) \times \big(FM_{ref}(7.0) - FM_{ref}(6.0)\big)$$

$$= 2.0 + 0.5 \times (2.4 - 2.0) = 2.2.$$

Table F: Median values of fat mass and fat-free mas estimated using anthropometric data from the Mexican Health and nutrition Survey 2018

Body mass index category	Age	**Males**		**Females**	
		Fat-free mass (kg)	Fat mass (kg)	Fat-free mass (kg)	Fat mass (kg)
Underweight	6	14.5	2.0	13.9	2.6
Underweight	7	16.4	2.4	16.6	3.0
Underweight	8	18.0	2.1	17.3	3.1
Underweight	9	19.3	2.4	17.5	2.9
Underweight	10	23.9	3.0	20.1	3.2
Underweight	11	23.8	2.7	24.0	3.9
Underweight	12	24.1	2.8	25.5	4.1
Underweight	13	28.3	3.0	32.7	5.4
Underweight	14	33.7	3.7	37.2	5.9

Underweight	15	35.7	4.5	32.3	5.2
Underweight	16	41.9	4.7	33.0	4.9
Underweight	17	42.7	4.8	31.6	4.8
Underweight	18	42.9	4.5	37.5	5.8
Normal weight	6	17.1	3.5	15.1	3.7
Normal weight	7	18.2	3.6	17.3	4.2
Normal weight	8	19.9	3.7	19.4	4.9
Normal weight	9	21.9	4.1	21.3	4.9
Normal weight	10	24.9	4.6	23.6	5.4
Normal weight	11	27.5	4.8	28.3	7.0
Normal weight	12	31.2	5.6	32.3	8.4
Normal weight	13	36.1	6.8	33.8	9.3
Normal weight	14	41.0	8.4	36.0	9.9
Normal weight	15	44.0	8.8	38.3	10.4
Normal weight	16	46.8	9.6	39.7	11.5
Normal weight	17	48.3	10.3	39.5	10.3
Normal weight	18	49.4	10.7	41.5	10.9
Overweight	6	19.2	4.6	17.7	5.7
Overweight	7	21.7	5.6	20.0	6.4
Overweight	8	24.6	5.9	22.2	7.0
Overweight	9	26.5	6.6	25.7	8.7
Overweight	10	29.9	8.1	29.6	10.6
Overweight	11	32.5	8.6	32.9	11.8
Overweight	12	37.8	10.2	36.7	14.7
Overweight	13	41.5	12.0	38.6	14.6
Overweight	14	48.0	15.3	41.0	16.2
Overweight	15	49.7	15.7	43.1	17.4
Overweight	16	53.3	18.4	45.7	19.8
Overweight	17	56.0	19.0	47.3	19.4
Overweight	18	56.7	18.9	46.0	19.2
Obesity	6	22.0	7.1	20.5	8.7
Obesity	7	25.0	8.1	23.4	9.3
Obesity	8	27.5	8.9	26.8	11.5
Obesity	9	30.9	10.8	29.3	12.8
Obesity	10	34.2	12.3	34.1	15.7
Obesity	11	38.2	14.5	37.6	17.4
Obesity	12	42.8	17.3	42.3	22.9
Obesity	13	48.1	21.0	43.5	26.7
Obesity	14	51.0	23.0	45.6	27.7
Obesity	15	54.9	25.5	48.1	28.1
Obesity	16	58.6	30.0	49.0	30.7
Obesity	17	58.4	27.2	50.7	30.0

Obesity	18	63.7	31.9	50.0	28.4

4 Intervention scenarios

To estimate the reduction in caloric intake we constructed three scenarios: 1) using Mexican data to inform the intervention effect, 2) using Mexican data to inform the intervention effect varying compliance, and 3) using meta-analytic data to inform the intervention scenario, and 4) using national data to inform the intervention effect by sex and socioeconomic status.

4.1 Using Mexican data to inform the intervention effect

For the main scenario and scenario 4 (sensitivity analysis), we identified non-essential energy-dense food and beverages (NEDFBs) based on the established guidelines in Mexico in 2020 for labeling processed foods and beverages with "excess of" calories, added sugars, fats, trans fat, or sodium. [16]. These guidelines are presented in Table G. For the main analysis, we estimated the consumption of NEDFBs inside schools stratified by type of school: elementary, junior high, and high school. For the sensitivity analysis 3, we stratified the NEDFBs inside school by sex and socioeconomic status.

Table G: General labeling specifications for prepackaged food and non-alcoholic beverages-Commercial and health information.

	**Specifications**
Supplemental nutritional information should be included on the label of prepackaged product that:<br>a) contain additives: free sugars, fats or sodium.<br>b) the energy value, the amount of free sugars, saturated fat, trans fat and sodium meet with the established nutritional profiles.	For solid foods per 100 grams: > 275 kcal total, > 10% of the total energy from free sugars, > 10% of the total energy from saturated fats, > 1% of the total from trans fat; for sodium> 350 mg/100g.<br>For liquids for every 100 milliliters:> 70 kcal total or >10 kcal of free sugars, > 10% of the total energy from free sugars, > 10% of the total energy from saturated fats, > 1% of the total from trans fat; for sodium> 45 mg (non-calorie drinks).
Definition of prepackaged	Prepackaged product with added free sugars are those in which free sugars have been added during the manufacturing process, and ingredients that contain added free sugars.<br>Added prepackaged products of fats are those in which vegetable or animal fats, partially hydrogenated vegetable oils or products and ingredients containing them have been added during the manufacturing process.<br>Added prepackaged sodium product are those in which any salt containing sodium or any ingredient containing added sodium has been used as an ingredient or additive during the manufacturing process.

4.2 Using Mexican data to the inform intervention effect, varying compliance

As a sensitivity analysis, we estimated the effect of compliance rate over the main scenarios (with and without energy compensation). As,the Food Frequency Questionnaire from ENSANUT 2018 does not include informa-

tion to distinguish school consumption in Mexico, we assumed that the compliance rate would directly affect the change in body weight:

$$Weight\ reduction_{compliance} \approx \%compliance \times Weight\ reduction_{main}\ . \tag{9}$$

For example, in the main scenario without energy compensation, we estimated an average weight reduction of 2.3 kg (see Table 1 in the main manuscript). That reduction was estimated assuming a compliance rate of 100%. Thus, under 80% of compliance, the expected weight would be estimated as 80% × 2.3 kg = 1.8 kg. To estimate reductions in obesity, we applied equation (9) for each individual in the sample and re-estimated obesity prevalence.

4.3 Using meta-analytic data to inform the intervention effect

This scenario is based on the meta-analysis performed by Micha et. al, evaluating interventions in the school food-environment. [17] We reviewed all the studies included in Micha's analysis and selected those that evaluated an intervention similar to ours (n =6), [18] ie, interventions that banned certain type of food and beverages. Table H shows the studies included in our analysis. Since the meta-analysis included the period March 2014 to December 2017, we performed a rapid review with the same search terms as Micha, et al., ("Effectiveness of school food environment policies on children's dietary behaviors: A systematic review and meta-analysis") from December 2017 until 2022, and we identified another study published in 2020 estimating kcal reduction after modifying the nutrition standards and banning processed foods and beverages inside the school. [19] Using a random-effect model, we obtained a total reduction in energy intake of 177.0 kcal (95% CI [-241.9, -112.2]) (**Pooled** in Table H). Given the heterogeneity of total energy intakes, we estimated the relative reduction from the pooled TEI of the control groups (1,811.0 kcal) –before the intervention or unexposed to the intervention. This reduction represented a change of -9.8% (95% CI [-13.4%, -6.2%]) from TEI. The studies were observational without a control group, so there is likely an overestimation of the effect, especially when compared to Ensanut estimates. Hence, we consider three effects: 100% (point estimate), 50%, and 25% of the effect.

Table H: Studies evaluating interventions in nutrition standards and banning processed foods and beverages inside schools. These studies were selected from the literature to estimate a meta-analytic parameter for the banning effect, based on Micha et al. (2018) meta-analytical analysis.

Study	Estimate	95% CI	Weight
Fung 2013[a]	-248.5	-301.2, -195.8	17.5
Spence 2014A Least[a]	-73.0	-143.0, -3.0	16.0
Spence 2014A Mid[a]	-162.0	-216.0, -108.0	17.4
Spence 2014A Most[a]	-253.0	-315.0, -191.0	16.7
Spence 2014B[a]	-259.0	-332.0, -186.0	15.8
Taber 2012[a]	-157.8	-371.0, 55.4	6.3
Turner 2020[b]	-5.8	-145.6, 134.1	10.3
**Pooled**	-177.0	-241.9, -112.2	100.0

[a]Selected from Micha et al. (2018) meta-analysis. [17]

[b]Turner et al. (2020) change in energy intake among US population. [19]

5 Microsimulation model

Body weight was simulated for each child or adolescent j in the data using the following equation: [10]

$$BW_j(t_{365}) = BW_j^{\text{model}}\big(age_j + t_{365};\ \text{sex}_j, FFM(t_0), FM(t_0), TEI\,(t_0, t_{365})\big) \tag{10}$$

where $t_0 = 0$ is the initial time (year = 2018), $t_{365} = 365$ (days; one year of simulation). $FFM(t_0)$ and $FM(t_0)$ are the initial (baseline) fat-free mass and fat mass of the child or adolescent, respectively, estimated with the

Deurenberg formula (equations (7) and (8)). $TEI\ (t_0, t_{365})$ is a vector containing daily energy intake from the initial time $t_0 = 0$ to the day $t_{365} = 365$, and depends on the scenarios considered in the analyses. For the business-as-usual scenario,

$$TEI\ (t_0, t_{365}) = (I_{ref}(0), I_{ref}(1), I_{ref}(2), \ldots, I_{ref}(d), \ldots, I_{ref}(365))\,, \tag{11}$$

$I_{ref}(0)$ and $I_{ref}(d)$ are the energy intake of reference at the inital time and at day d, respectively, as estimated in section 3. For the intervention scenarios, we estimated the daily intake at day d considering the corresponding reduction:

$$I_{\text{scenario}}\ (d) = (\%\ intake\ change) \times I_{ref}(d), \tag{12}$$

where $\%\ intake\ change = (1 - \%\ reduction)$ for the main scenario without compensation and the sensitivity using a meta-analytic parameter, and $\%\ intake\ change = \%\ compensation \times (1 - \%\ reduction)$ for the main scenarios with compensation.

Thus, in the intervention scenarios,

$$TEI\ (t_0, t_{365}) = (I_{\text{scenario}}\ (0), I_{\text{scenario}}\ (1), \ldots, I_{\text{scenario}}\ (d), \ldots, I_{\text{scenario}}\ (365))\,. \tag{13}$$

5.1 Summary of Hall et al. equations

From Hall et al. [10], we know that:

- $EB(t) = I(t) + E(t)$;
- $E(t) = K + \gamma_{FFM} FFM + \gamma_{FM} FM + \beta \Delta I(t) + \delta BW(t) + \eta_{FFM} \frac{dFFM}{dt} + \eta_{FM} \frac{dFM}{dt}$;
- $BW(t) = FFM(t) + FM(t)$;
- $\frac{dFFM}{dt} = \dfrac{p(I - E) + g}{\rho_{FFM}}$;
- $\frac{dFM}{dt} = \dfrac{(1 - p)(I - E) - g}{\rho_{FM}}$

For our analysis, we assumed no trend in energy intake ($\Delta I = 0$). Then, we estimated the energy intake of reference (no intervention) as

$$\begin{aligned}
I_{ref} &= EB_{ref} + E_{ref} \\
&= EB_{ref} + K + \gamma_{FFM} FFM_{ref} + \gamma_{FM} FM_{ref} + 0 + \delta BW_{ref} + \eta_{FFM} \frac{dFFM}{dt} + \eta_{FM} \frac{dFM}{dt} \\
&= EB_{ref} + K + \gamma_{FFM} FFM_{ref} + \gamma_{FM} FM_{ref} + 0 + \delta BW_{ref} + \eta_{FFM} \frac{p(EB_{ref}) + g}{\hat{\rho}_{FFM}} + \eta_{FM} \frac{(1-p)(EB_{ref}) - g}{\rho_{FM}} \\
&= EB_{ref} + K + (\gamma_{FFM} + \delta) FFM_{ref} + (\gamma_{FM} + \delta) FM_{ref} + \frac{\eta_{FFM}}{\hat{\rho}_{FFM}} (pEB_{ref} + g) + \frac{\eta_{FM}}{\rho_{FM}} ((1-p)EB_{ref} - g)
\end{aligned}$$

6 Model validation

The original body weight model was calibrated using the reference body composition data from Mexican children presented in section 3.2.1. As a form of validation, we compared simulated average body weights of children aged 6-17, with observed average body weights of children 7-18 from the ENSANUT 2018 (Figures B and C). The simulated body weights were obtained from one year of simulation without intervention; we excluded individuals who changed their baseline BMI category. Our one-year predictions were consistent with the observed average weights by nutritional status for the corresponding ages in the ENSANUT survey, with average errors of 2.23 kg in underweight status, 0.56 kg in normal weight status, 1.22 kg in overweight status, 1.32 kg in obesity status and an overall error of 0.83 kg.

Figure B: Comparison of mean body weight between the Dynamics of Childhood Growth and Obesity Model and mean observed values in ENSANUT 2018 according to nutritional status in children from 6 to 17 years old

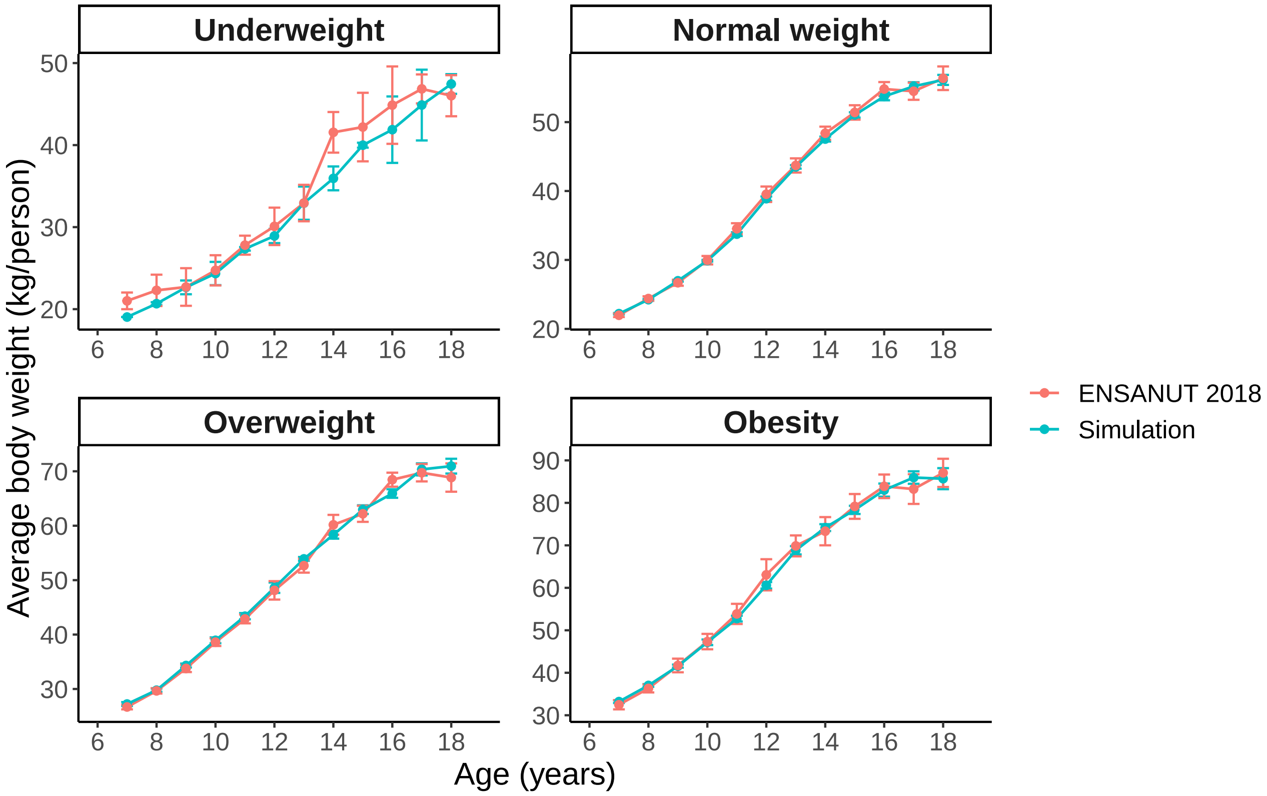

Figure C: Comparison of mean body weight between the Dynamics of Childhood Growth and Obesity Model and mean observed values in ENSANUT 2018 in children from 6 to 17 years old

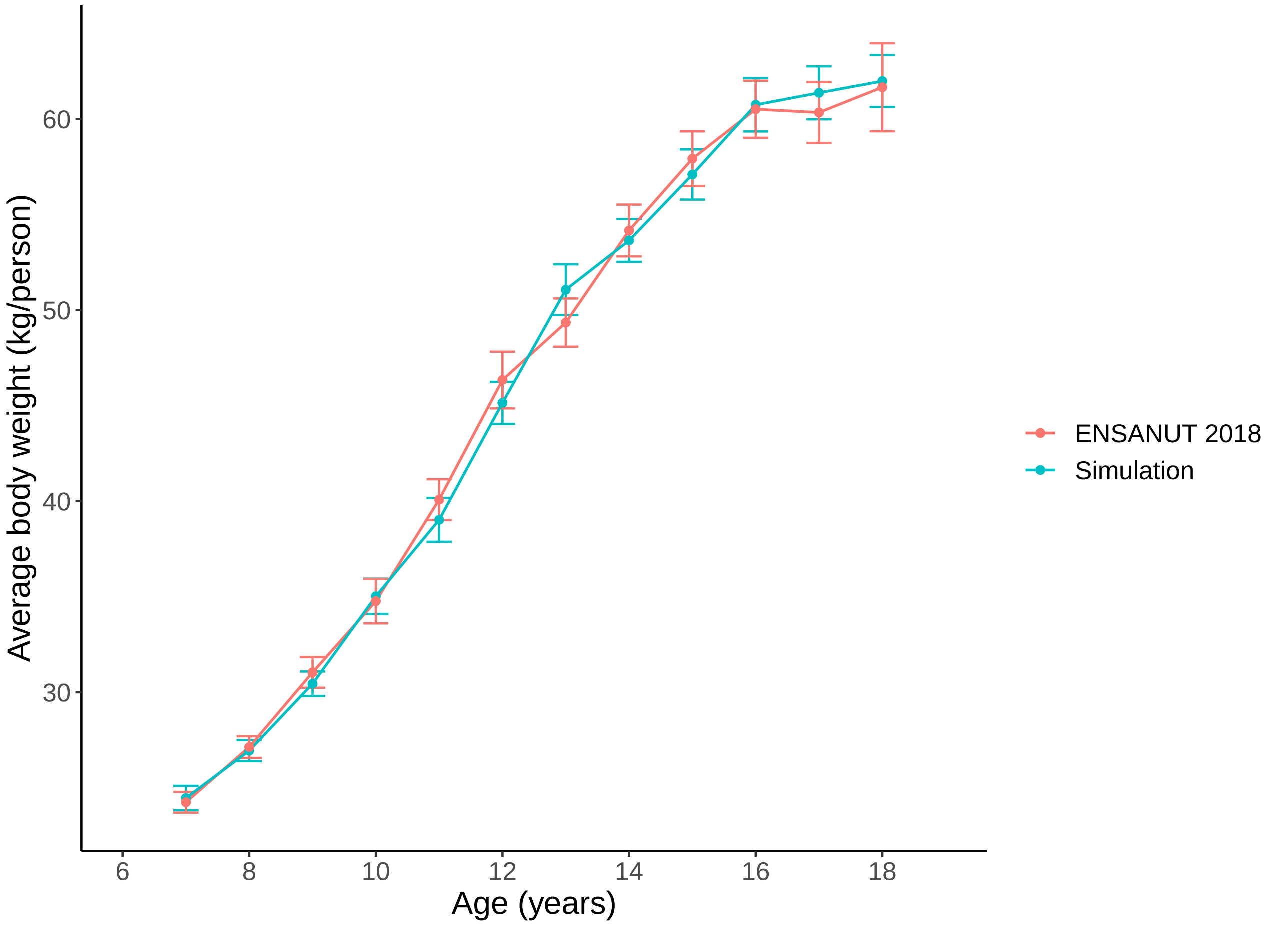

7 Baseline vs Business as usual characteristics

Table I: Estimates of energy intake, body weight, BMI, and obesity for the baseline and after one year without intervention (BAU).

	**Total (UI*)**	
	**Baseline**	**BAU**
Energy intake (kcal/person/day)	2246 (2234, 2234)	2316 (2304, 2328)
Body weight (kg/person)	48.2 (47.6, 47.6)	51.1 (50.6, 51.6)
Body mass index (kg/m2)	21.0 (20.9, 20.9)	21.5 (21.3, 21.7)
Obesity (pp)	16.6 (15.4, 15.4)	16.1 (16.1, 16.1)

*UI = Uncertainty interval

8 Uncertainty intervals

We estimated uncertainty intervals by generating 1,000 Monte Carlo simulations of the results, assuming normal distributions for the parameters considered in each scenario. For the scenario with and without energy

compensation, we varied the % TEI from non-essential energy dense foods and beverages consumed at schools. For the scenario with energy compensation, we also varied the percentage of energy compensation by generating the change in regulated foods, change in non-regulated foods, change in regulated beverages, and change in non-regulated beverages. For the sensitivity using meta-analytical data, we simulated the uncertainty for the meta-analytical parameter, which represented the reduction in TEI estimated due to the ban. For the sensitivity analysis considering the ban effect by sex and socioeconomic status (SES), we varied the % TEI from non-essential energy-dense foods and beverages consumed at schools. The parameters described above were summarized in Table C. Uncertainty from the DCGO model, proposed by Hall et al., was not assessed due to computational time but in future projects, it could be done by simulating some model parameters.

9 Data available online

The database and a description of the variables considered for the analyses are available here (data.dta). All results were estimated considering the survey design (syntax in R with *survey package* [20–22]):

```
Svy.design.2018 <- svydesign(id = ~ 1, strata = ~est_dis , PSU = ~upm_dis ,
                   weights = ~svy_weights , data = data)

options(survey.lonely.psu = ''adjust'')
```

The database consists of children between 6 to 18 years old considered for estimating the median reference fat mass and fat-free mass presented in Table F (n = 10, 412). Those median values were estimated using the estimated baseline fat mass and fat-free mass (variables: *initial_fm*, and *initial_ffm*), and a rounded age in years (*age_years* := *round(age_months/12)*). Age in months (*age_months*) was not used for the median estimations because of the lack of sample in some groups of age, sex and bmi categories. However, age in months (*age_months*) was used for estimating body weight with Hall's model, and also Zscores for bmi and height. To replicate the main results using the database, set *flag_main_results* = 1 (n = 9,754, children between 6-17 years old). The file "Total energy intake by day.csv" includes the vector of total energy intake estimated for each children in "data.dta" (rows = individuals, columns = TEI by day).

References

[1] Secretaría de Educación Pública; Secretaría de Salud. ACUERDO mediante el cual se establecen los lineamientos generales para el expendio y distribución de alimentos y bebidas preparados y procesados en las escuelas del Sistema Educativo Nacional. México: Diario Oficial de la Federación;; 2014. Available from: https://www.dof.gob.mx/nota_detalle.php?codigo=5344984&fecha=16/05/2014#gsc.tab=0.

[2] Secretaría de Economía. Modificación a la Norma Oficial Mexicana NOM-051-SCFI/SSA1-2010, Especificaciones generales de etiquetado para alimentos y bebidas no alcohólicas preenvasados-Información comercial y sanitaria, publicada el 5 de abril de 2010. México: Diario Oficial de la Federación; 2020. Available from: https://www.dof.gob.mx/normasOficiales/8150/seeco11_C/seeco11_C.html.

[3] Habicht JP. [Standardization of quantitative epidemiological methods in the field]. Boletin de la Oficina Sanitaria Panamericana Pan American Sanitary Bureau. 1974 5;76(5):375-84.

[4] WHO Multicentre Growth Reference Study Group. WHO child growth standards: length/height-for-age, weight-for-age, weight-for-length, weight-for-height and body mass index-for-age: methods and development. Geneva: World Health Organization; 2006. Available from: https://www.who.int/publications/i/item/924154693X.

[5] de Onis M, Onyango AW, Borghi E, Siyam A, Nishida C, Siekmann J. Development of a WHO growth reference for school-aged children and adolescents. Bulletin of the World Health Organization. 2007 9;85(9):660-7.

[6] Shamah-Levy T, Vielma-Orozco E, Heredia-Hernández O, Romero-Martínez M, Mojica-Cuevas J, Cuevas-Nasu L, et al.. Encuesta Nacional de Salud y Nutrición 2018-19: Resultados Nacionales;. Available from: https://ensanut.insp.mx/encuestas/ensanut2018/doctos/informes/ensanut_2018_informe_final.pdf.

[7] Gutiérrez JP, Rivera-Dommarco J, Shamah-Levy T, Villalpando-Hernández S, Franco A, Cuevas-Nasu L, et al. Encuesta Nacional de Salud y Nutrición 2012. Resultados Nacionales. 2012.

[8] Taillie LS, Bercholz M, Popkin B, Reyes M, Colchero MA, Corvalán C. Changes in food purchases after the Chilean policies on food labelling, marketing, and sales in schools: a before and after study. The Lancet Planetary Health. 2021 8;5(8):e526-33.

[9] Romero-Martínez M, Shamah-Levy T, Vielma-Orozco E, Heredia-Hernández O, Mojica-Cuevas J, Cuevas-Nasu L, et al. Encuesta Nacional de Salud y Nutrición 2018-19: metodología y perspectivas. Salud Pública de México. 2019 12;61(6, nov-dic):917-23.

[10] Hall KD, Butte NF, Swinburn BA, Chow CC. Dynamics of childhood growth and obesity: development and validation of a quantitative mathematical model. The Lancet Diabetes & Endocrinology. 2013 10;1(2):97-105.

[11] Hugues Y, Díaz-Zavala RG, Quizán-Plata T, Corvalán C, Haby MM. Poor compliance with school food environment guidelines in elementary schools in Northwest Mexico: A cross-sectional study. PLOS ONE. 2021 11;16(11):e0259720.

[12] Deurenberg P, Weststrate JA, Seidell JC. Body mass index as a measure of body fatness: age- and sex-specific prediction formulas. British Journal of Nutrition. 1991 3;65(2):105-14.

[13] Katan MB, de Ruyter JC, Kuijper LDJ, Chow CC, Hall KD, Olthof MR. Impact of Masked Replacement of Sugar-Sweetened with Sugar-Free Beverages on Body Weight Increases with Initial BMI: Secondary Analysis of Data from an 18 Month Double–Blind Trial in Children. PLOS ONE. 2016 7;11(7):e0159771.

[14] Nelson KM, Weinsier RL, Long CL, Schutz Y. Prediction of resting energy expenditure from fat-free mass and fat mass. The American Journal of Clinical Nutrition. 1992 11;56(5):848-56.

[15] Forbes GB. Lean Body Mass-Body Fat Interrelationships in Humans. Nutrition Reviews. 2009 4;45(10):225-31.

[16] Romero Anaya, Raúl and Novelo Baeza, José Alonso. PROYECTO de Modificación a la Norma Oficial Mexicana NOM-051-SCFI/SSA1-2010, Especificaciones generales de etiquetado para alimentos y bebidas no alcohólicas preenvasados-Información comercial y sanitaria, publicada el 5 de abril de 2010; 2019. Available from: https://www.dof.gob.mx/nota_detalle.php?codigo=5575205&fecha=11/10/2019.

[17] Micha R, Karageorgou D, Bakogianni I, Trichia E, Whitsel LP, Story M, et al. Effectiveness of school food environment policies on children's dietary behaviors: A systematic review and meta-analysis. PLOS ONE. 2018 3;13(3):e0194555.

[18] Fung C, McIsaac JLD, Kuhle S, Kirk SFL, Veugelers PJ. The impact of a population-level school food and nutrition policy on dietary intake and body weights of Canadian children. Preventive Medicine. 2013 12;57(6):934-40.

[19] Turner L, Leider J, Piekarz-Porter E, Chriqui JF. Association of State Laws Regarding Snacks in US Schools With Students' Consumption of Solid Fats and Added Sugars. JAMA Network Open. 2020 1;3(1):e1918436.

[20] R Core Team. R: A Language and Environment for Statistical Computing. Vienna, Austria: R Foundation for Statistical Computing; 2021. Available from: `https://www.R-project.org/`.

[21] Lumley T. Complex Surveys: A Guide to Analysis Using R: A Guide to Analysis Using R. John Wiley and Sons; 2010.

[22] Lumley T. survey: analysis of complex survey samples; 2020.
