# Supplementary material for: Predicted impact of banning nonessential, energy-dense food and beverages in schools in Mexico: A microsimulation study
Source: PLoS Med. 2024 May 10;21(5):e1004394. doi: 10.1371/journal.pmed.1004394 (PMC11086919; doi:10.1371/journal.pmed.1004394)
Supplement: Attachment — Submitted filename: Response to reviewers. Banning.docx [file pmed.1004394.s002.docx]

Dear Dr. Barrientos-Gutierrez,

Thank you very much for submitting your manuscript "Expected impact of banning non-essential, energy-dense food and beverages in schools in Mexico: a microsimulation study" (PMEDICINE-D-23-03317R1) for consideration at PLOS Medicine.

Your paper was evaluated by a senior editor and discussed among all the editors here. It was also discussed with an academic editor with relevant expertise, and sent to independent reviewers, including a statistical reviewer. The reviews are appended at the bottom of this email and any accompanying reviewer attachments can be seen via the link below:

In light of these reviews, I am afraid that we will not be able to accept the manuscript for publication in the journal in its current form, but like to consider a revised version that addresses the reviewers' and editors' comments. We cannot make any decision about publication until we have seen the revised manuscript and your response, and we plan to seek re-review by one or more of the reviewers.

In revising the manuscript for further consideration, your revisions should address the specific points made by each reviewer and the editors. Please also check the guidelines for revised papers at <http://journals.plos.org/plosmedicine/s/revising-your-manuscript> for any that apply to your paper. In your rebuttal letter you should indicate your response to the reviewers' and editors' comments, the changes you have made in the manuscript, and include either an excerpt of the revised text or the location (eg: page and line number) where each change can be found. Please submit a clean version of the paper as the main article file; a version with changes marked should be uploaded as a marked up manuscript.

In addition, we request that you upload any figures associated with your paper as individual TIF or EPS files with 300dpi resolution at resubmission; please read our figure guidelines for more information on our requirements: <http://journals.plos.org/plosmedicine/s/figures>. While revising your submission, please upload your figure files to the PACE digital diagnostic tool, <https://pacev2.apexcovantage.com/>. PACE helps ensure that figures meet PLOS requirements. To use PACE, you must first register as a user. Then, login and navigate to the UPLOAD tab, where you will find detailed instructions on how to use the tool. If you encounter any issues or have any questions when using PACE, please email us at PLOSMedicine@plos.org.

We expect to receive your revised manuscript by Feb 19 2024 11:59PM. Please email us (plosmedicine@plos.org) if you have any questions or concerns.

***Please note while forming your response, if your article is accepted, you may have the opportunity to make the peer review history publicly available. The record will include editor decision letters (with reviews) and your responses to reviewer comments. If eligible, we will contact you to opt in or out.***

We ask every co-author listed on the manuscript to fill in a contributing author statement, making sure to declare all competing interests. If any of the co-authors have not filled in the statement, we will remind them to do so when the paper is revised. If all statements are not completed in a timely fashion this could hold up the re-review process. If new competing interests are declared later in the revision process, this may also hold up the submission. Should there be a problem getting one of your co-authors to fill in a statement we will be in contact. YOU MUST NOT ADD OR REMOVE AUTHORS UNLESS YOU HAVE ALERTED THE EDITOR HANDLING THE MANUSCRIPT TO THE CHANGE AND THEY SPECIFICALLY HAVE AGREED TO IT. You can see our competing interests policy here: <http://journals.plos.org/plosmedicine/s/competing-interests>.

Please use the following link to submit the revised manuscript:

<https://www.editorialmanager.com/pmedicine/>

Your article can be found in the "Submissions Needing Revision" folder.

Please ensure that the paper adheres to the PLOS Data Availability Policy (see <http://journals.plos.org/plosmedicine/s/data-availability>), which requires that all data underlying the study's findings be provided in a repository or as Supporting Information. For data residing with a third party, authors are required to provide instructions with contact information for obtaining the data. PLOS journals do not allow statements supported by "data not shown" or "unpublished results." For such statements, authors must provide supporting data or cite public sources that include it.

We look forward to receiving your revised manuscript.

Sincerely,

Katrien Janin, PhD

PLOS Medicine

[plosmedicine.org](http://plosmedicine.org/)

-----------------------------------------------------------

Comments from the Academic Editor:

1. For the meta-analysis, I'm surprised by an intervention that reduces total energy intake by 10% - this is substantial and I think unrealistic. But I think that the authors do a good job of not over-emphasising this finding from sensitivity analysis. It would be nice to have some discussion of how realistic the estimation from the meta-analysis really is. It just seems unlikely to me that children would reduce energy intake by 10%long term.

We agree with the editor that the meta-analysis could overestimate the effect of the intervention, as stated in methods and discussion. The meta-analysis includes only observational studies (mostly before-after and cross-sectional studies), which could overestimate the true effect due to lack of a suitable control and confounding. We have added an additional caution note in both the methods and discussion sections to highlight this. Also, studies included in the meta-analysis were from high-income countries, where a better infrastructure and organization for school food services could improve compliance with respect to middle- and low-income countries, so we are also highlighting this too. Finally, we included a more comprehensive description on how we conducted the systematic review for the meta-analysis as follows:

Methods section at point 3 (page 5):

*As the included studies were observational* ***(mostly before-after and cross-sectional studies)****, we expected an overestimation of the banning effect compared to experimental designs* ***due to the lack of a suitable control group and potential confounding****.*

Discussion, fourth paragraph:

*Our sensitivity analysis using a meta-analytical input, showed a much higher expected effect of the intervention;* ***however, studies included were observational, often lacked a control group and were subject to residual confounding. These limitations could explain the large estimated effects.* Also, *studies in the meta-analysis were conducted in high-income countries, where infrastructure and organization of school food services is better and where compliance could also be higher than in middle- and low-income countries.***

2. The paper is poorly contextualized. I'd like to see more discussion of: limitations of meta-analysis as above, why compliance with the existing regulations is low and why it might be expected to improve with the new regulations (compliance seems the key to the whole paper - both in terms of the justification for the intervention and results of sensitivity analysis),

Thank you for the comment. As suggested, we made several modifications in the introduction and discussion to contextualize why compliance will be improved with the updated law, as follows:

Introduction, third paragraph:

*In 2010, Mexico implemented national nutritional guidelines* ***as a part of the Sectoral Health Program 2007-2012****[19] to regulate* ***the preparation, sale and distribution of foods and beverages that exceeded recommended nutritional standards in elementary and middle schools*** *[20]. By 2014,* ***through a reform of the General Education Law,*** ***these guidelines*** *became mandatory and punishable* *[21]****. The law provided an exception for Fridays, when sales of all products were allowed (supplementary table 1)****. T****his law was poorly implemented; between 2012 and 2015,*** *only 27% of food sold in schools met the nutritional criteria [22]. In 2018 in Northwest Mexico only 15% of schools had fully implemented the guidelines [23].* ***Low compliance has been explained by the poor dissemination and knowledge about the guidelines, lack of monitoring, enforcement and sanctions, failure to ban junk food advertising, and not applying restrictions to venues near schools*** *[22,23]****.*** *Valuable lessons were learned from this experience: 1) increase parental involvement, 2) offer healthy substitutes for popular foods and beverages, 3) engage food delivery staff and teachers, 4) provide free drinking water, and 5) regulate advertising of unhealthy food in and around schools [22,23].*

Introduction, fourth paragraph:

***In December 2023, the Mexican Senate approved a reform to strengthen the General Education Law*** *[24]****. This reform law implied a structural change in the regulatory framework, emphasizing direct government involvement in assuring compliance. The approved legislation mandates the Secretary of Education to issue new General Guidelines for the preparation, sale and distribution of prepared and processed foods and beverages in schools. Guidelines are under development, but the main changes to the law include the ban on advertising unhealthy food, expand the law to venues near schools, standardize the nutritional criteria to front of package labels proposed in the General Law of Health (Art 212) (supplementary table 2)*** *[25]****,*** *promote healthier food alternatives prioritizing plain water* ***and stronger sanctions for non-compliance.*** ***The Secretary of Education will now assume the responsibility of monitoring and ensuring the enforcement of the new guidelines through school directors and supervisors, replacing the previous role of parental committees.*** *It is expected the law reform will promote better compliance, in the US, policies restricting SSB consumption in schools had higher impact when mandatory and when an administrative agency was designated as responsible for its implementation[26]. We aimed to estimate the expected impact of banning sales of non-essential energy-dense food and beverages (NEDFBs) in schools on total caloric intake and obesity prevalence of school-aged children and adolescents one year after its implementation, considering varying degrees of compliance.*

Discussion, fourth paragraph:

*The school context is very important for implementing and impacting health interventions. Our sensitivity analysis using a meta-analytical input, showed a much higher expected effect of the intervention;* ***however, studies included were observational, often lacked a control group and were subject to residual confounding. These limitations could explain the large, estimated effects.*** ***Also****, studies in the meta-analysis were conducted in high-income countries, where infrastructure and organization of school food services is better and where compliance could also be higher than in Mexico.* ***Compliance will play a key role in the success of any law targeting the school food environment. Previous experience with the guidelines issued in 2010 showed that the lack of dissemination and training, the lack of regulations outside and near schools, and the lack of monitoring were a key element in their low compliance [22,23]. With the issuance of the new law, these limitations are addressed. Ensuring a mechanism of implementation, evaluation and monitoring will be key to improve compliance. Our sensitivity analysis shows that if all schools comply with the law, the intervention is predicted to reduce 0.8 kg/person, but if only 30% of schools comply with it, the intervention is predicted to reduce 0.2 kg/person.***

4. how the impacts of this intervention compare to other potential approaches to obesity (there is a little bit of this, but I'd suggest expanding further).

Thank you for your comment. Comparing with other interventions is a challenging task, since we have to restrict all comparisons to a similar age group. We revised recently published studies to see if we could add something to the studies already mentioned; we only found one pre-print that estimated the empirical impact of front-of-pack labeling using an online survey in adolescents. The study has important limitations and it is difficult to compare their results with ours, but it is the only evidence from other Mexican interventions in this age group. We added as follows:

*Our prediction of -33 kcal/person/day (UI [-42, -25] kcal/person/day) from banning NEDFBs, which translates into a -0.8 kg (UI [-1.0, -0.6] kg), is higher than the expected impact of the 10% SSBs tax and 8% junk food tax on Mexican children, but lower than both tax interventions combined. Taxes on SSBs and junk food were expected to reduce 17.6 kcal/person (UI [16.3, 18.8] kcal/person) and 17.4 kcal/person (UI [16.4, 18.4] kcal/person), respectively, one year after their implementation [33,39]. This translates into body weight reductions of 0.42 kg (UI [0.39, 0.45] kg) for the SSBs tax and 0.40 kg (UI not available) for the junk food tax. We used a similar microsimulation model, a similar age group, and targeted the same food as both previous studies, but with a different intervention (tax vs. school ban). In all three cases, weight reduction is simulated for one year. Our nonlinear simulation model suggests that further impacts on body weight after the first year of simulation are yet to be expected [40].* ***Empirical evidence from an online survey in adolescents aged 13-17 years in Mexico, showed a reduction of 38.7% in the purchase of NEDFBs after the implementation of the front-of-package labeling.[41] However, the relative reduction does not allow us to quantify the reduction in kcal to properly compare the results with our study.***

5. Related, I'd also like to see the UI in table 2 - it's not clear at what point the effect is indistinguishable from null.

Thank you for the comment. As suggested, we included the UIs in Table 2:

Comments from the Editorial Team:

We would like to ask the authors the following:

i) In line with the academic editor and reviewer comments, please improve the contextualisation of the presented research

Thank you for the comment. As suggested, we made several modifications in the introduction to improve contextualization.

ii) for the ‘Sensitivity scenario using meta-analytic data to inform the intervention effect’ in the method section please detail your search criteria and search period, inclusion and exclusion criteria and the basis upon which you selected studies to be included in the meta analysis. We note that studies were mainly sourced from ‘Effectiveness of school food environment policies on children’s dietary behaviors: A systematic review and metaanalysis.’ publication. We also note you have added one study, but how was this study sourced? Did you perform a systematic literature review to update the Micha study? This is unclear to us. Please provide more details (beyond those details in Section 3.3 in S1 Appendix) and ask you to move pertinent information into the main manuscript. We also like more details on how you arrived at the estimated 177-kcal reduction (9.8% reduction in TEI), especially given that this substantially differs from the data derived from ENAUD.

Thank you for your comment. We added more details from the meta-analysis and the relative reduction in TEI in the appendix.

**4.3 Sensitivity scenario using meta-analytic data to inform the intervention effect.**

T*his scenario is based on the meta-analysis performed by Micha et. al, evaluating interventions in the school food-environment. [17] We reviewed* ***all*** *the studies included in Micha’s analysis and selected those that evaluated an intervention similar to ours* ***(n=6)****, [18] i.e., interventions that banned certain types of food and beverages.* ***Since the meta-analysis included the period March 2014 to December 2017, we performed a rapid review with the same search terms as Micha, et al., (“Effectiveness of school food environment policies on children’s dietary behaviors: A systematic review and meta-analysis”) from December 2017 until 2022, and we identified*** *another study* ***published in 2020*** *estimating kcal reduction after modifying the nutrition standards and banning processed foods and beverages inside the school. [19]*

*Using a random-effect model, we obtained a reduction in total energy intake of 177.0 kcal (95% CI [-241.9, -112.2]) (Pooled in Table* ***H****).* ***Given the heterogeneity of total energy intakes, we estimated the relative reduction from the pooled TEI of the control groups (1,811.0 kcal)*** *–before the intervention or unexposed to the intervention.* ***This reduction represented a change of -9.8% (95%CI [-13.4%, -6.2%]) from TEI. The studies were observational without a control group, so*** *there is likely an overestimation of the effect,* ***especially when compared to Ensanut estimates****.* ***Hence,*** *we consider three effects: 100% (point estimate), 50%, and 25% of the effect.*

In the discussion, please add more details on why these estimates differ so much, and how realistic this scenario (or not) may be. Once again, we appreciate you have added that this is likely an overestimate, but ask a bit more detail and ask why you think this model - if likely an overestimate - adds to the discussion).

We have added a note to both the methods section and discussion as to why the meta-analysis results are likely to be an overestimate (see response to comment 1 of the Academic Editor). Despite this limitation, we included it as a sensitivity analysis because it is based on the best available data from the results of real-world implementation of similar interventions. It also draws attention to the lack of real-world evaluations of this type of policy intervention and the need for better information to inform future models and policies.

iii) In the Discussion, be more explicit about how the effect sizes compare to prior studies; and contextualize findings including with regard to compensatory calorie intake and weight regain.

Thank you for your comment. We included a paragraph with two prior observational studies that evaluated similar interventions. However, both are observational studies, including inherently energy compensation and weight regain. Still, they did not measure TEI, so we cannot quantify compensation nor weight regain. The main limitations for comparison are the nature of the studies, observational versus simulation. We modified the text in the discussion as follows:

*The expected impact on BMI of banning NEDFBs in schools in our analysis is higher compared to prior studies evaluating similar interventions.* ***The comparison is difficult because other studies are observational.*** *We predicted a BMI change of -0.3 kg/m2 (UI [-0.4, -0.2] kg/m2) for children aged 6 to 17 years, compared to -0.06 kg/m2 (95% CI [-0.11, -0.01] kg/m2) for a similar mandatory school-based intervention implemented in six Canadian provinces for children aged 12 to 15 years [36]. They also estimated a yearly reduction of 0.06 kg/m2 (95% CI [0.01, 0.11] kg/m2) to reach 0.32 kg/m2 (95% CI [0.03, 0.62]) after 5 to 8 years of intervention [36]. Differences between the yearly model and our one-year simulation model is linearity. The Canadian model assumes the intervention increases linearly over time; we assumed a non-linear function of calories (Hall’s TEI equation) on body mass over time, and that a significant proportion (around 50%) of the intervention effect will be achieved during the first year and then decrease progressively (Hall’s weight change model is also non-linear) [37]. A Brazilian study showed reductions in body composition among girls aged 11 to 15 years, although the results are expressed in BMI z-scores for age [38], hindering comparisons. However, the direction of the effect is the same in response to regulations on the sale of processed food and beverages in schools*. **B*oth observational studies and our simulation results showed that banning NEDFBs in schools should lead to a reduction in BMI, the magnitude of the effect relies on different settings for the observational studies and different assumptions of our simulation model (i.e., compliance rate and compensation).***

iv) In the Discussion, also clearly point out what this adds to prior knowledge and say something about what the findings are useful for (and what they are not useful for), and expand on what these findings mean beyond Mexico.

We have added the following text to the discussion section as paragraph six:

***Our modeling is based on the best available evidence of the potential impact of the new law. It suggests that an important impact on obesity prevalence can be expected if the law is implemented and enforced as intended, but that lower effects will be expected if enforcement is suboptimal. Our study highlights the need for better evidence about the actual impact of government laws and policies such as these to determine their effectiveness and whether adjustments are needed. If the predicted impact of the new law is achieved it could serve as an example beyond Mexico of how to achieve real changes in obesity. However, this will also depend on what other laws and policies are in place to prevent overweight and obesity.***

GENERAL:

Please provide 95% CIs and p values for all results where appropriate (including the abstract), check and amend throughout. We suggest reporting statistical information in the following format: ‘x’; (95% CI [‘y’,’ z’] p value) and use commas as opposed to hyphens (as these can be confused with negative values) to separate upper and lower bounds. For p values, please report as p<0.001 and where higher as 'p=0.002'. Please add the statistical method used to your method section. We also invite you to report p values consistently to the third decimal digit - thousandths.

Reflecting the probabilistic nature of the model, we have added uncertainty intervals to our results. We do not have statistical methods to calculate 95% confidence intervals or p-values from the model. We included the suggested format of the intervals. Some examples are presented below:

In the abstract:

*We found that children could reduce their daily energy intake by 33 kcal/day/person (uncertainty interval,* ***UI, [25, 42] kcal/day/person****), reducing on average* *0.8 kg/person* ***(UI [0.6, 1.0] kg/person****) and 1.5 percentage points (pp) in obesity* ***(UI [1.1, 1.9] pp)*** *one year after implementing the law.*

As you suggested, we also included details about the uncertainty estimation in the S1	Appendix:

***Uncertainty intervals***

***We estimated uncertainty intervals by generating 1,000 Monte Carlo simulations of the results, assuming normal distributions for the parameters considered in each scenario. For the scenario with and without energy compensation, we varied the % TEI from non-essential energy dense foods and beverages consumed at schools. For the scenario with energy compensation, we also varied the percentage of energy compensation by generating the change in regulated foods, change in non-regulated foods, change in regulated beverages, and change in non-regulated beverages. For the sensitivity using meta-analytical data, we simulated the uncertainty for the meta-analytical parameter, which represented the reduction in TEI estimated due to the ban. For the sensitivity considering the ban effect by sex and socioeconomic status (SES), we varied the % TEI from non-essential energy dense foods and beverages consumed at schools. The parameters described above were summarized in Table C. Uncertainty from the DCGO model, proposed by Hall et al., was not assessed due to computational time but in future projects it could be done by simulating some model parameters.***

STUDY DESIGN: MODELLING STUDIES

Of all authors who submit a modelling study we ask for inclusion of specific items, derived from Geoffrey P Garnett, Simon Cousens, Timothy B Hallett, Richard Steketee, Neff Walker. Mathematical models in the evaluation of health programmes. (2011) Lancet DOI:10.1016/S0140-6736(10)61505-X.

Please ensure all the items listed below are included with your manuscript. Please review the list below and confirm/revise as necessary:

i) Please provide a diagram that shows the model structure, including how the disease natural history is represented, the process and determinants of disease acquisition, and how the putative intervention could affect the system.

We modified Figure 1, as the model representation with all stages of the modeling process (estimation of energy intake, considered scenarios, simulation of body weight, and estimation of the intervention effect):

***Figure 1. Step-by-step modeling process, showing inputs and outputs of the model.*** *Changes in body weight and obesity prevalence are estimated by comparing the intervention scenario with BAU one year after the intervention.* ****TEI****(t_0_, t_365_ ) is a vector with the daily total energy intakes from t_0_ to t_365_ = (t_0_+365), considering sex, age, baseline (initial) BMI category, Fat Mass (FM) and Fat-Free Mass (FFM). FM y FFM were estimated using the body fat percentage equation (%BF) developed and validated by Deurenberg et al.: FM = %BF*BW=(1.51*BMI - 0.7*Age - 3.6*Sex + 1.4)*BW/100 and FFM = BW - FM, where BW denote body weight and BMI represents body mass index. ** SES = Socioeconomic status.*

ii) Please provide a complete list of model parameters, including clear and precise descriptions of each parameter, together with the values or ranges for each, with justification or the primary source cited, and important caveats about the use of these values noted.

Thank you for the comment. We reviewed and verified that Table C in the S1 Appendix fulfills the requirements:

iii) Please provide a clear statement about how the model was fitted to the data [including goodness-of-fit measure, the numerical algorithm used, which parameter varied, constraints imposed on parameter values, and starting conditions].

Thank you for your comment. We verified that the main text include the description of the modeling structure considered in the analyses:

Methods

*Briefly, we first estimated baseline fat-free mass (FFM) and fat mass (FM) for the children and adolescents based on body mass index, age, and sex, using an equation developed and validated by Deurenberg, et al [27]. This equation estimates the body fat percentage for children and adolescents, derived from a multiple linear regression. Baseline FFM and FM were used to predict the total energy intake per day (TEI) depending on sex, age and BMI category using a non-linear function proposed by Hall et al. [28]. This TEI function considered children’s growth and was derived from the sum of two components: energy balance (= energy intake - energy expenditure), and energy expenditure. We estimated the effect of the law on TEI in Mexican children under three different scenarios: 1) main scenario, using Mexican data to inform the intervention effect, 2) sensitivity scenario varying law compliance, and 3) sensitivity scenario, using meta-analytic data to inform the effect of the intervention.* *Considering a business-as-usual scenario (BAU; no intervention) and the estimated intervention effect on TEI, we used Hall´s weight change microsimulation model to estimate the potential impact on body weight and obesity prevalence of children and adolescents that could be achieved one year after implementing the intervention in Mexican schools.*

We summarized the model inputs, considering standard deviations, in Table C in S1 Appendix:

In the S1 Appendix, we included the algorithm to estimate uncertainty intervals:

***Uncertainty intervals***

***We estimated uncertainty intervals by generating 1,000 Monte Carlo simulations of the results, assuming normal distributions for the parameters considered in each scenario. For the scenario with and without energy compensation, we varied the % TEI from non-essential energy dense foods and beverages consumed at schools. For the scenario with energy compensation, we also varied the percentage of energy compensation by generating the change in regulated foods, change in non-regulated foods, change in regulated beverages, and change in non-regulated beverages. For the sensitivity using meta-analytical data, we simulated the uncertainty for the meta-analytical parameter, which represented the reduction in TEI estimated due to the ban. For the sensitivity considering the ban effect by sex and socioeconomic status (SES), we varied the % TEI from non-essential energy dense foods and beverages consumed at schools. The parameters described above were summarized in Table C. Uncertainty from the DCGO model, proposed by Hall et al., was not assessed due to computational time but in future projects it could be done by simulating some model parameters.***

iv) For uncertainty analyses, please state the sources of uncertainties quantified and not quantified [can include parameter, data, and model structure].

We included the description of the uncertainty analysis in the S1 Appendix as follows:

***Uncertainty intervals***

***We estimated uncertainty intervals by generating 1,000 Monte Carlo simulations of the results, assuming normal distributions for the parameters considered in each scenario. For the scenario with and without energy compensation, we varied the % TEI from non-essential energy dense foods and beverages consumed at schools. For the scenario with energy compensation, we also varied the percentage of energy compensation by generating the change in regulated foods, change in non-regulated foods, change in regulated beverages, and change in non-regulated beverages. For the sensitivity using meta-analytical data, we simulated the uncertainty for the meta-analytical parameter, which represented the reduction in TEI estimated due to the ban. For the sensitivity considering the ban effect by sex and socioeconomic status (SES), we varied the % TEI from non-essential energy dense foods and beverages consumed at schools. The parameters described above were summarized in Table C. Uncertainty from the DCGO model, proposed by Hall et al., was not assessed due to computational time but in future projects it could be done by simulating some model parameters.***

v) Please provide sensitivity analyses to identify which parameter values are most important in the model. Uncertainty estimates seek to derive a range of credible results on the basis of an exploration of the range of reasonable parameter values. The choice of method should be presented and justified.

Thank you for the comment. We considered three sensitivity scenarios accounting for (1) compliance of the law, (2) alternative intervention effect based on observational studies, (3) socioeconomic status and sex. In this review we added another sensitivity analysis to stratify results by socioeconomic status and sex. These scenarios were described in the “Methods” section:

***2. Sensitivity scenario varying compliance****. In the previous scenario, we assumed that all Mexican schools would implement the intervention appropriately. However, an evaluation of the implementation of the 2014 NEDFBs regulation guideline showed that only 15% of schools fully complied in one Mexican State.[23] Due to the inclusion of enforcement and sanction mechanisms for the new law, we expect a higher compliance rate of at least twice the 2014 observed value (30%), with the possibility of reaching 100% depending on enforcement. For this analysis, we assumed that the compliance rate directly modifies weight reduction:*

$$Weight reduction=Compliance rate X Weight reduction$$

***3. Sensitivity scenario using meta-analytic data to inform the intervention effect.*** *To estimate the effect of the law, we conducted a meta-analysis based on published observational studies that implemented similar interventions, i.e., nutrition standards or banning NEDFBs in schools, compared to control. We calculated the mean difference in habitual intake (kcal/day) using a random effects model and used it to determine the percentage reduction in TEI. Section 4.3 in S1 Appendix details the included studies and the meta-analytic estimation. We estimated a 177-kcal reduction, meaning a 9.8% reduction in TEI (95% CI [-13.4, -6.2] %) in children and adolescents after the intervention. As the included studies were observational (mostly before-after and cross-sectional studies), we expected an overestimation of the banning effect compared to experimental designs due to the lack of a suitable control group and potential confounding. Therefore, we considered three possible scenarios:*

a. *100% of the effect (-9.8% TEI),*
b. *50% of the effect (-4.9% TEI),*
c. *25% of the effect (-2.45% TEI).*

***4.*** ***Sensitivity scenario using Mexican dietary data to inform the intervention effect by socioeconomic status and sex.***

***For this scenario, the NEDFBs energy contribution was estimated by socioeconomic status (SES) and sex to assess the potential equity impact of the policy. As in the main scenario, we used the 24-hour dietary recall data from ENSANUT 2012 to estimate the energy contribution of NEDFBs to TEI in schools and considered both the energy compensation and no energy compensation assumptions. For females, NEDFBs intake in schools represented about 4.7%, 5.0%, and 6.5% of the TEI for the low, middle and high SES, respectively. For males, those values were about 2.3%, 3.5%, and 4.7% of the TEI for the low, middle and high SES, respectively.***

vi) Please discuss the scientific rationale for this choice of model structure and identify points where this choice could influence conclusions drawn. Please also describe the strength of the scientific basis underlying the key model assumptions.

Thank you for the comment. The scientific rationale for the choice of the model structure and the scientific basis underlying the key model assumptions were presented in the “Methods” and “Discussion” sections, as follows:

**Methods**

*Briefly, we first estimated baseline fat-free mass (FFM) and fat mass (FM) for the children and adolescents based on body mass index, age, and sex, using an equation developed and validated by Deurenberg, et al [27]. This equation estimates the body fat percentage for children and adolescents, derived from a multiple linear regression. Baseline FFM and FM were used to predict the total energy intake per day (TEI) depending on sex, age and BMI category using a non-linear function proposed by Hall et al. [28]. This TEI function considered children’s growth and was derived from the sum of two components: energy balance (= energy intake - energy expenditure), and energy expenditure. We estimated the effect of the law on TEI in Mexican children under three different scenarios: 1) main scenario, using Mexican data to inform the intervention effect, 2) sensitivity scenario varying law compliance, and 3) sensitivity scenario, using meta-analytic data to inform the effect of the intervention.* *Considering a business-as-usual scenario (BAU; no intervention) and the estimated intervention effect on TEI, we used Hall´s weight change microsimulation model to estimate the potential impact on body weight and obesity prevalence of children and adolescents that could be achieved one year after implementing the intervention in Mexican schools.*

*Simulation of body weight after one year of intervention*

*Individual’s body weight was simulated under both BAU and intervention scenarios using the Dynamics of Childhood Growth and Obesity Model (DCGO), proposed by Hall, et al.[28] Briefly, the DCGO is a non-linear model that estimates changes in body weight over time (t) using a system of two differential equations to predict fat mass (FM(t)) and fat free mass (FFM(t)). To predict body weight (BW(t)), we considered the relation between energy intake rate and energy expenditure, adjusted by a growth term. All these variables consider children´s characteristics (sex, age, initial height, and body weight), and other parameters that account for complex physiological processes during childhood and adolescence. The DCGO model was previously validated with experimental weight data [28] and calibrated to the Mexican population. In this study, we re-calibrated and validated the model using Mexican data from the ENSANUT 2018 [33]. An overview of the microsimulation model and the calibration process is presented in sections 5 and 6 in S1 Appendix.*

*Assumptions and other parameters*

*We assumed that all children and adolescents attend school and that the contribution of NEDFBs consumed at school remains constant over time. According to Hall’s microsimulation model, physical activity progressively decreases with age.[28] For the BAU scenario, we assumed children’s growth as the only factor for TEI daily changes without considering any previous trends or externalities that could cause a change in TEI. Hence, no increase in obesity prevalence is considered over one year of simulation. Additionally, we assumed that the height of children and adolescents would change according to WHO’s height-for-age curves*.

Discussion

*Some limitations must be acknowledged. First, the total impact of the intervention may be underestimated because we are using 24-h nutritional recall data from 2012 to estimate the calories from junk food consumed at school, which is known to underestimate unhealthy food consumption. Also, for children under 12 years of age, 24-h nutritional recall data obtains information from parents or guardians, who may not be well informed about their child’s consumption patterns at school. Still, ENSANUT is representative at the national level, allowing us to make population estimates generalizable to the Mexican context. Results may be overestimated because we are assuming that all children and adolescents go to school, but in the 2019-2020 cycle, 98.7% of 6–12-year-olds, 95.9% of 13–15-year-olds and 75.5% of 16-18-year-olds were enrolled in school [42]. Given the uncertainty of the compliance rate, we tested different compliance levels, but we assumed that it would be at least twice the compliance of the 2014 guidelines implemented in Mexico (15%) [23]. We are assuming that the banning effect and compliance rate are equal for public or private schools. Evidence exists that private schools had better compliance with the regulation than public schools [23], but the overall effect is likely to be small, as most Mexican children attend public schools[42]. Overestimation could also arise from energy compensation, either from allowed food and beverages in school or through NEDFBs consumed outside of schools. We considered substantial levels of food compensation (81%) and beverages (39%), based on household-level estimates after the Chilean policies on food labeling, marketing, and sales in schools [32]. Another study from Chile after the same policies did not report caloric compensation, but compensation for specific nutrients. They reported an 80% sugar compensation for children, but complete sugar compensation for adolescents [17]. Our model considers a growth-related increase in TEI (Table I in S1 Appendix) but does not include an increase in TEI due to past obesity trends over time. However, this assumption should have little effect on estimates, given the one-year span of our simulation. Finally, we are not considering the potential effect of banning junk food near schools, as our study only included the expected impact inside schools.*

TITLE: We suggest you replace ‘expected’ by ‘predicted’ (Predicted impact of banning non-essential, energy-dense food and beverages in schools in Mexico: a microsimulation study).

We changed the title as suggested.

AUTHORS SUMMARY: At this stage, we ask that you include a short, non-technical Author Summary of your research to make findings accessible to a wide audience that includes both scientists and non-scientists. The Author Summary should immediately follow the Abstract in your revised manuscript. This text is subject to editorial change and should be distinct from the scientific abstract. Please see our author guidelines for more information: <https://journals.plos.org/plosmedicine/s/revising-your-manuscript#loc-author-summary>

Ideally each sub-heading should contain 2-3 single sentence, concise bullet points containing the most salient points from your study.

In the final bullet point of ‘What Do These Findings Mean?’ Please include the main limitations of the study in non-technical language.

**Why was this study done?**

- **School-based interventions have been recognized as effective policies to improve nutritional knowledge and reduce obesity-related diseases.**
- **In December 2023, the Chamber of Representatives approved an amendment that strengthens and updates the General Education Law and the nutrition guidelines to ban the sales and advertising of non-essential energy-dense food and beverages (NEDFBs) in schools.**

**What did the researchers do and find?**

- **The intervention was predicted using four different scenarios: 1) using national data on NEDFBs consumption in schools by education level, 2) varying law compliance, and 3) using pooled effect on caloric change with observational studies and 4) stratifying results by socioeconomic status and sex.**
- **We used age-specific equations to predict baseline fat-free mass and fat mass and total energy intake per day. We used Hall´s microsimulation model to predict body weight and obesity prevalence of children and adolescents one year after implementing the intervention in Mexican schools.**
- **If the law is implemented and enforced as designed, children could reduce their daily energy intake by 33 kcal/day/person, reducing on average 0.8 kg/person and 9% in obesity one year after implementing the law.**
- **Considering 50% of the law compliance in schools, the intervention effect could reduce body weight on average by 0.4 kg/person. If compliance is only 30%, the predicted impact would be 0.2 kg/person.**

**What do these findings mean?**

- **Our modeling study suggests that an important impact on obesity prevalence can be expected if the law is implemented and enforced as intended.**
- **The results of this study rely on (1) the compliance of the schools, (2) not considering historical trends on obesity or NEDFBs consumed in schools during one year of simulation, (3) considering only the ban impact inside schools, excluding effects near and outside schools.**
- **If successful, this law could serve as an example beyond Mexico on how to achieve changes in obesity in children.**

DISCUSSION:

Please present and organize the Discussion as follows: a short, clear summary of the article's findings; what the study adds to existing research and where and why the results may differ from previous research; strengths and limitations of the study; implications and next steps for research, clinical practice and/or public policy implications; followed by a one-paragraph conclusion. Please remove all subheadings within your Discussions

Thanks for the comment. We removed all subheading within discussion. We have re-organized discussion as follows: first paragraph a short, clear summary of the article's findings; second and third paragraph what the study adds to existing research and where and why the results may differ from previous research (second paragraph describes similar interventions, different contexts, and third paragraph describes different interventions, similar contexts and similar methodology). Limitations paragraph is now 4th paragraph; 5th paragraph includes the implications of the policy and the importance of school compliance. The 6th paragraph was added as a reviewer recommendation about the outreach of the study and implications for other countries and the 7th conclusion.

6th paragraph

***Our modeling is based on the best available evidence of the potential impact of the new law. It suggests that an important impact on obesity prevalence can be expected if the law is implemented and enforced as intended, but that lower effects will be expected if enforcement is suboptimal. Our study highlights the need for better evidence about the actual impact of government laws and policies such as these to determine their effectiveness and whether adjustments are needed. If the predicted impact of the new law is achieved it could serve as an example beyond Mexico of how to achieve real changes in obesity. However, this will also depend on what other laws and policies are in place to prevent overweight and obesity.***

-----------------------------------------------------------

Comments from the reviewers:

Reviewer #1: The manuscript "Expected impact of banning non-essential, energy-dense food and beverages in schools in Mexico: a microsimulation study" describes the expected effect of a new law (which is currently in the legislative process) banning the sale and advertising of unhealthy foods inside schools and surrounding, in Mexico. Using previously proposed equations for estimating total daily energy intake, and nationally representative data on body weight, weight status and intake of unhealthy foods/beverages at schools, authors modeled the effect of the policy on energy intake, body weight, body mass index and prevalence of obesity among school-age children, considering different scenarios (i.e., dietary compensation, school compliance, effect size of the decrease in energy intake). Simulating the effect of a policy which is under legislative discussion is a good strategy to inform policy-makers' decision, and a good example of the potential use of scientific

investigation when it is well conducted, in a relevant topic, and timely done/published. Moreover, policies aiming at improving school food environments are an especially relevant strategy to address the obesity pandemic. Therefore, this manuscript is highly relevant.

1. The manuscript is well written, the methodology is clearly explained and the details are presented largely in the supplementary file; results are clear and relevant. The document reflects a well conducted analysis. From my perspective, authors fail in presenting the current situation of school food environments in the introduction, and contrasting it with the new scenario (the one expected after the policy implementation). I believe one difference between the current and the expected situation is that in the former nutritional standard is optional, whereas the latter implies a mandatory situation (i.e., a ban).

Additionally, currently the standards apply to preparation, sale and distribution of foods, whereas the new scenario will apply just to food sales and marketing (is there a food feeding program in Mexico?)

Thank you for your comment. We consider it pertinent to clarify that the implementation of the guidelines was mandatory since their update in 2014. However, it had some deficiencies, particularly in relation to the monitoring mechanisms and sanctions for non-compliance. On the other hand, the constitutional reform accepted in December 2023 aims to issue updated guidelines that will also regulate the preparation, sale and distribution with a clearer and stricter surveillance and punishment framework directed by school authorities. We clarify both points as suggested and modify the introduction in third and fourth paragraph as follows:

Introduction, third paragraph:

*In 2010, Mexico implemented national nutritional guidelines* ***as a part of the Sectoral Health Program 2007-2012****[19] to regulate* ***the preparation, sale and distribution of foods and beverages that exceeded recommended nutritional standards in elementary and middle schools*** *[20]. By 2014,* ***through a reform of the General Education Law,*** ***these guidelines*** *became mandatory and punishable* *[21]****. The law provided an exception for Fridays, when sales of all products were allowed (supplementary table 1)****. T****his law was poorly implemented; between 2012 and 2015,*** *only 27% of food sold in schools met the nutritional criteria [22]. In 2018 in Northwest Mexico only 15% of schools had fully implemented the guidelines [23].* ***Low compliance has been explained by the poor dissemination and knowledge about the guidelines, lack of monitoring, enforcement and sanctions, failure to ban junk food advertising, and not applying restrictions to venues near schools*** *[22,23]****.*** *Valuable lessons were learned from this experience: 1) increase parental involvement, 2) offer healthy substitutes for popular foods and beverages, 3) engage food delivery staff and teachers, 4) provide free drinking water, and 5) regulate advertising of unhealthy food in and around schools [22,23].*

Introduction, fourth paragraph:

***In December 2023, the Mexican Senate approved a reform to strengthen the General Education Law*** *[24]****. This reform law implied a structural change in the regulatory framework, emphasizing direct government involvement in assuring compliance. The approved legislation mandates the Secretary of Education to issue new General Guidelines for the preparation, sale and distribution of prepared and processed foods and beverages in schools. Guidelines are under development, but the main changes to the law include the ban on advertising unhealthy food, expand the law to venues near schools, standardize the nutritional criteria to front of package labels proposed in the General Law of Health (Art 212) (supplementary table 2)*** *[25]****,*** *promote healthier food alternatives prioritizing plain water* ***and stronger sanctions for non-compliance.*** ***The Secretary of Education will now assume the responsibility of monitoring and ensuring the enforcement of the new guidelines through school directors and supervisors, replacing the previous role of parental committees.*** *It is expected the law reform will promote better compliance, in the US, policies restricting SSB consumption in schools had higher impact when mandatory and when an administrative agency was designated as responsible for its implementation [26]. We aimed to estimate the expected impact of banning sales of non-essential energy-dense food and beverages (NEDFBs) in schools on total caloric intake and obesity prevalence of school-aged children and adolescents one year after its implementation, considering varying degrees of compliance.*

Finally, I believe the nutritional criteria for identifying unhealthy foods are different between both scenarios, given -I understood- the new regulation uses the

same cutoffs used for defining foods that should have a warning label, which were developed in the last few years. All these aspects should be clearly stated in the introduction (of course, I might be wrong in my interpretation).

Thank you for the comment. As suggested, we modify the fourth paragraph of the introduction to clarify that the new regulation uses the same cutoffs used for defining foods that should have a warning label, likewise, we include a comparative table in the appendix with the nutritional criteria of the 2014 guidelines and those considered in the last constitutional reform:

Introduction, fourth paragraph:

***In December 2023, the Mexican Senate approved a reform to strengthen the General Education Law*** *[24]****. This reform law implied a structural change in the regulatory framework, emphasizing direct government involvement in assuring compliance. The approved legislation mandates the Secretary of Education to issue new General Guidelines for the preparation, sale and distribution of prepared and processed foods and beverages in schools. Guidelines are under development, but the main changes to the law include the ban on advertising unhealthy food, expand the law to venues near schools, standardize the nutritional criteria to front of package labels proposed in the General Law of Health (Art 212) (supplementary table 2)*** *[25]****,*** *promote healthier food alternatives prioritizing plain water* ***and stronger sanctions for non-compliance.*** ***The Secretary of Education will now assume the responsibility of monitoring and ensuring the enforcement of the new guidelines through school directors and supervisors, replacing the previous role of parental committees.*** *It is expected the law reform will promote better compliance, in the US, policies restricting SSB consumption in schools had higher impact when mandatory and when an administrative agency was designated as responsible for its implementation [26]. We aimed to estimate the expected impact of banning sales of non-essential energy-dense food and beverages (NEDFBs) in schools on total caloric intake and obesity prevalence of school-aged children and adolescents one year after its implementation, considering varying degrees of compliance.*

Specific comments

The quality of Figure 1 should be improved.

Thank you for your comment. As you suggested, we improved the quality of Figure 1.

***Figure 1. Step-by-step modeling process, showing inputs and outputs of the model.*** *Changes in body weight and obesity prevalence are estimated by comparing the intervention scenario with BAU one year after the intervention.* ****TEI****(t_0_, t_365_ ) is a vector with the daily total energy intakes from t_0_ to t_365_ = (t_0_+365), considering sex, age, baseline (initial) BMI category, Fat Mass (FM) and Fat-Free Mass (FFM). FM y FFM were estimated using the body fat percentage equation (%BF) developed and validated by Deurenberg et al.: FM = %BF*BW=(1.51*BMI - 0.7*Age - 3.6*Sex + 1.4)*BW/100 and FFM = BW - FM, where BW denote body weight and BMI represents body mass index. ** SES = Socioeconomic status.*

"BAU" should be presented earlier in the text, the first time when "business as usual" is mentioned.

Thank you for the comment. The first time that "business as usual" was mentioned was in the methods section.

***Considering a business-as-usual scenario (BAU; no intervention)*** *and the estimated intervention effect on TEI, we used Hall´s weight change microsimulation model to estimate the potential impact on body weight and obesity prevalence of children and adolescents that could be achieved one year after implementing the intervention in Mexican schools.*

Data from ENSANNUT 2012 were used for estimating the dietary share of unhealthy foods eaten at schools, whereas ENSANUT 2018 was used for obtaining the weight status data and estimating total energy intake; please provide the rationale for doing so.

Thank you for your comment. We used ENSANUT 2012 to estimate the energy contribution of foods and beverages prohibited inside schools because this survey has 24-H recall data on consumption inside the school; We used a food frequency questionnaire from ENSANUT 2018 to estimate total energy intake, but this questionnaire does not have specific data on consumption inside schools. We modify the methods section accordingly to clarify why we use 2012 data, as follows:

1. ***Main scenario using Mexican dietary data to inform the intervention effect.*** *We used the 24-hour dietary recall data from ENSANUT 2012 [30] to estimate the energy contribution of NEDFBs to TEI inside schools, since* ***this survey obtained information on place of consumption.*** *NEDFBs were identified using the guidelines established in Mexico in 2020 for “excess'' calories, added sugars, fats, trans fats, or sodium (Table G in S1 Appendix) [31]. According to ENSANUT 2012, NEDFBs intake in schools represents 4.2%, 5.4%, and 4.1% of the TEI in elementary, junior high, and high school students, respectively. Table C in S1 appendix shows the contributions with the confidence intervals.*

Authors estimated a compensation scenario based on compensation data from household food purchases obtained after implementing the Chilean food labelling regulation. A different article evaluating the Chilean regulation showed a greater compensatory effect (for energy from total sugars and saturated fats) for the dietary improvement obtained at schools among adolescents (dietary compensation made at home and other locations): <https://ijbnpa.biomedcentral.com/articles/10.1186/s12966-023-01445-x>. Given total energy intake is not reported in the article, data cannot be used for the current analyses; however, authors could discuss these data, acknowledging a potential underestimation of the dietary compensation.

Thank you for your comment and the reference. We looked at it and for sugar the compensation in children was 80% and for adolescents 100% three years after the implementation. The 80% compensation is in line with what we used for food, but they found no effect for adolescents with respect to sugar. We added it to the discussion as follows:

*Some limitations must be acknowledged. First, the total impact of the intervention may be underestimated because we are using 24-h nutritional recall data from 2012 to estimate the calories from junk food consumed at school, which is known to underestimate unhealthy food consumption. Also, for children under 12 years of age, 24-h nutritional recall data obtains information from parents or guardians, who may not be well informed about their child’s consumption patterns at school.* ***Still, ENSANUT is representative at the national level, allowing us to make population estimates generalizable to the Mexican context****. Results may be overestimated because we are assuming that all children and adolescents (100%) go to school, but in the 2019-2020 school cycle, 98.7% of 6-12 year olds, 95.9% of 13-15 year olds and 75.5% of 16-18 year olds were enrolled in school.[42] Given the uncertainty of the compliance rate, we tested different compliance levels, but we assumed that it would be at least twice the compliance of the 2014 guidelines implemented in Mexico (15%).[23] We are assuming that the banning effect and compliance rate are equal for public or private schools. Evidence exists that private schools had better compliance with the regulation than public schools [23], but the overall effect is likely to be small, as most Mexican children attend public schools [42]. Overestimation could also arise from* ***energy compensation, either from allowed food and beverages in school or through NEDFBs consumed outside of schools. We considered substantial levels of food compensation (81%) and beverages (39%), based on household-level estimates after the Chilean policies on food labeling, marketing, and sales in schools[32]. Another study from Chile after the same policies did not report caloric compensation, but sugar compensation. They reported an 80% sugar compensation for children, but complete sugar compensation for adolescents [17]. Our model considers a growth-related increase in TEI (Table I in S1 Appendix) but does not include an increase in TEI due to past obesity trends over time. However, this assumption should have little effect on estimates, given the one-year span of our simulation.*** *Finally, we are not considering the potential effect of banning junk food near schools, as our study only included the expected impact inside schools.*

2. Legend could be improved for Figure 2 (the squares are too little to see the colors).

Thank you for the comment. We increased the size of the squares. We also removed the “75% of the meta-analytic effect” scenario from the figure since it was not part of our methodology. Instead, we included the “25% of the meta-analytic effect” scenario, which had been described in the methods. Also, we corrected the uncertainty intervals for the meta-analytic scenarios, which did not consider the effect on the standard deviation.

3. The second paragraph of discussion reads: "The Canadian model assumes the intervention increases linearly over time; our model assumes a non-linear function of calories on body mass over time, and a significant proportion (around 50%) of the intervention effect will be achieved during the first year and then decrease progressively." I am confused with this statement. I assume "our model" refers to the one used in the current analysis; in that case, it is relevant the non-linear simulation model is presented earlier in the text and in a clearer way.

Thank you for your comment. Yes, "our model" referred to the one used in the analysis. As you suggested, we rewrite the text to improve clarity.

In the method section,

*Figure 1 summarizes each step of the modeling process. Briefly, we first estimated baseline fat-free mass (FFM) and fat mass (FM) for the children and adolescents based on body mass index, age, and sex, using equations developed and validated by Deurenberg, et al [27].* ***This equation estimates the body fat percentage for children and adolescents, derived from a multiple linear regression.*** ***Baseline FFM and FM were used to predict the total energy intake per day (TEI) depending on sex, age and BMI category using a non-linear function proposed by*** *Hall et al. [28].* ***This*** ***TEI function considered children’s growth and was derived from the sum of two components: energy balance (= energy intake - energy expenditure), and energy expenditure.*** *We estimated the effect of the law on TEI in Mexican children under three different scenarios: 1) main scenario, using Mexican data to inform the intervention effect, 2) sensitivity scenario varying law compliance, and 3) sensitivity scenario, using meta-analytic data to inform the effect of the intervention. Considering a business-as-usual scenario (BAU; no intervention) and the estimated intervention effect on TEI, we used Hall´s weight change microsimulation model to estimate the potential impact on body weight and obesity prevalence of children and adolescents that could be achieved one year after implementing the intervention in Mexican schools. A more detailed description of the modeling process is presented in the next subsections and in S1 appendix. Model inputs are presented in Table C in S1 Appendix.*

*Estimated total energy intake by day (TEI)*

*We estimated the daily TEI from day t0 to day t365 (one-year simulation period) for each individual using a* ***non-linear*** *equation proposed by Hall et al.[28] This equation accounts for daily increases in energy intake due to children’s growth, dependent on sex, age, and baseline height and body weight. Hall’s TEI equation is presented in S1 Appendix, section 3.*

*Simulation of body weight after one year of intervention*

*Individual’s body weight was simulated under both BAU and intervention scenarios using the Dynamics of Childhood Growth and Obesity Model (DCGO), proposed by Hall, et al.[28]. Briefly,* ***the DCGO is a non-linear*** *model* ***that*** *estimates changes in body weight over time (t) using a system of two differential equations to predict fat mass (FM(t)) and fat free mass (FFM(t)). To predict body weight (BW(t)), we considered the relation between energy intake rate and energy expenditure, adjusted by a growth term. All these variables consider children´s characteristics (sex, age, initial height, and body weight), and other parameters that account for complex physiological processes during childhood and adolescence. The DCGO model was previously validated with experimental weight data[28], and calibrated to the Mexican population. In this study, we re-calibrated and validated the model using Mexican data from the ENSANUT 2018[33]. An overview of the microsimulation model and the calibration process is presented in sections 5 and 6 in S1 Appendix.*

In the discussion section,

*The Canadian model assumes the intervention increases linearly over time;* ***we*** *assumed a non-linear function of calories* ***(Hall’s TEI equation)*** *on body mass over time, and that a significant proportion (around 50%) of the intervention effect will be achieved during the first year and then decrease progressively* ***(Hall’s weight change model is also non-linear).***

4. In the third paragraph, authors commented on modeling analyses performed previously for Mexican taxes on foods and sugary-sweetened beverages. It would be relevant to discuss whether these estimations were confirmed with real data. If this is not the case, this would be a good opportunity to discuss about the considerations to be considered to better interpret simulation analyses.

Thank you for your comment. Empirical evidence of the impact of taxes on health outcomes is limited. In Mexico, a recent study focused on adolescents from urban areas, found a decrease (-1.3 percentage points) in the prevalence of girls at risk of being overweight or obese in cities where the prices of SSB’s change exceeded 10% after tax implementation. However, no significant associations were found among boys. We acknowledge the relevance of further research considering cross-sectional/longitudinal data to evaluate changes in weight-related outcomes linked to national interventions aimed at improving the health of the Mexican population.

We added a paragraph in discussion to talk about the considerations of simulation analysis as also suggested by a reviewer, as follows:

***Our modeling is based on the best available evidence of the potential impact of the new law. It suggests that an important impact on obesity prevalence can be expected if the law is implemented and enforced as intended. The modeling also serves as a baseline against which the actual effects of the implementation of the law can be measured, once observational studies exist. Our study highlights the need for better evidence about the actual impact of government laws and policies such as these to determine their effectiveness and whether adjustments are needed. If the predicted impact of the enforcement of the law is achieved, it could serve as an example beyond Mexico of how to achieve real changes in obesity. However, this will also depend on what other laws and policies are in place to prevent overweight and obesity.***

Reviewer #2: This study addresses the critical issue of childhood obesity in Mexico by evaluating the potential impact of a government proposal to ban the sale and advertising of non-essential energy-dense foods and beverages (NEDFBs) in and around schools. Aimed at school-aged children and adolescents (6-17 years), the study utilizes age-specific predictive equations for baseline fat-free and fat mass to estimate total daily energy intake (TEI). Incorporating data from national sources and meta-analytic evidence, the research explores three scenarios: one reflecting the intervention's effect based on national data, another considering varying levels of law compliance, and a third informed by meta-analytic data on caloric impact. The study employs Hall's microsimulation model to forecast changes in body weight and obesity prevalence within a year of the law's potential implementation. Findings suggest a reduction in daily TEI by 33 kcal, translating to an average weight loss

of 0.8 kg per person and a 1.5 percentage point drop in obesity prevalence. These effects could vary with compliance rates; higher compliance may enhance the intervention's effectiveness. In conclusion, the ban could significantly curtail obesity among Mexican youth, with adherence to the law being a crucial determinant of success. Overall, this is a well-designed the conducted study. The methodology employed is robust, and the significance of the findings is pertinent for guiding policy decisions and advancing the health of children and adolescents in Mexico. Here are my detailed comments on the paper.

1. Please add line numbers for easier reference

Done.

2. Introduction: While the introduction effectively outlines the situation in Mexico, it would benefit from a brief comparison with global trends or similar initiatives in other countries. This could provide a broader context, showing how Mexico's situation is unique or similar to global trends. The authors can consider adding a few sentences comparing Mexico's obesity rates, school-based interventions, and legislative measures with those of other countries facing similar challenges. This comparison could highlight the uniqueness of Mexico's approach or align it with global best practices.

Thank you for your comment. We added more information about similar initiatives implemented in Chile, considering that both Mexico and Chile are pioneers in this kind of policies worldwide, we could expect that the results would be similar in terms of health and nutrition outcomes. A sentence was added in paragraph 2 in introduction, as follows:

*School-based interventions have been identified as effective policies to influence children's food consumption, nutritional knowledge, and obesity-related behaviors [9,10]. School-based interventions, such as nutritional standards for food and beverages sold in schools, have shown pooled reductions in unhealthy food intake of -0.17 servings/day and in sugar-sweetened beverages (SSBs) intake of -0.18 servings/day, resulting in reductions of -49 and -79 kcal/day in schools, respectively [11]. Countries such as Australia, Bermuda, Chile, France, and Slovenia have implemented restrictions on the accessibility of junk food and sugary beverages within school facilities, including banning vending machines, implementing nutritional guidelines to improve school meals, and promoting water and milk as the only beverage options [12–16].* ***Particularly in Latin America, Chile implemented a package of obesity prevention initiatives that included front-of-package warning labels, child-directed food advertisement bans, and school regulations. Three years after the intervention, total sugars consumed by children at school decreased 11.8 pp, saturated fats decreased 1.1 pp and sodium decreased 10.3 mg/100 kcal. In adolescents, total sugars and saturated fats consumed at school decreased 5.3 pp and 1.5 pp respectively [17]. In 2022 the Chilean Ministry of Education reported that, school-age children decreased their prevalence of obesity by 4.8% relative to 2021[18], which suggests promising results for countries implementing similar interventions, such as Mexico.***

3. Introduction: The introduction discusses the legislative changes and their potential impact on obesity rates among school-aged children, but it doesn't explicitly mention how these changes are perceived by key stakeholders such as parents, teachers, and the children themselves. Understanding their perspectives could add depth to the study, as these groups are directly affected by such policies.

Thank you for your comment. Currently there is little evidence based on qualitative studies that evaluate the perception of key stakeholders regarding the implementation of the 2014 guidelines. The available literature reflects that the main barriers identified are the lack of -dissemination, -knowledge and -interest in knowing the guidelines from parents, school directors and vendors inside schools. We include in the third paragraph of the introduction as follows:

*In 2010, Mexico implemented national guidelines* ***as a part of the Sectoral Health Program 2007-2012****[19] to regulate* ***the preparation, sale and distribution of foods and beverages that exceeded recommended nutritional standards in elementary and middle schools****[20]. By 2014,* ***through a reform of the General Education Law these guidelines became mandatory and punishable*** *[21].* ***The law provided an exception for Fridays, when sales of all products were allowed (supplementary table 1). This law was poorly implemented; between 2012 and 2015****, only 27% of food sold in schools met the nutritional criteria [22]. In 2018 in Northwest Mexico only 15% of schools had fully implemented the guidelines [23].* ***Low compliance has been explained by the poor dissemination and knowledge about the guidelines, lack of monitoring, enforcement and sanctions, failure to ban junk food advertising, and not applying restrictions to venues near schools*** *[22,23]. Valuable lessons were learned from this experience: 1) increase parental involvement, 2) offer healthy substitutes for popular foods and beverages, 3) engage food delivery staff and teachers, 4) provide free drinking water, and 5) regulate advertising of unhealthy food in and around schools [22,23].*

However, we recognize that the perception of the changes in the Education law by the key stakeholders is an area of opportunity for future research.

4. Introduction: The authors could include a brief discussion on potential implementation challenges. This could encompass issues such as the availability of healthier food options, logistical considerations in changing school canteens, budget constraints, and the need for ongoing monitoring and enforcement.

Thank you for your comment. As suggested, we included some relevant points in the third paragraph of introduction that we consider will be the main challenges in the proper implementation of the new law in the fourth paragraph as follows:

***Guidelines are under development, but the main changes to the law include the ban on advertising unhealthy food, expand the law to venues near schools, standardize the nutritional criteria to front of package labels proposed in the General Law of Health (Art 212) (supplementary table 2)[25], promote healthier food alternatives prioritizing plain water and stronger sanctions for non-compliance. Also, to enhance compliance, the Secretary of Education will now monitor and ensure the enforcement of the new guidelines through school directors and supervisors, replacing the previous role of parental committees.***

5. Method: When the authors reference the equations developed by Deurenberg and by Hall for predicting fat-free mass, fat mass, and daily total energy intake at the beginning of the method section, it would be beneficial to include concise descriptions of these methods. Since readers may not be familiar with these specific equations, providing a brief overview of how they were developed, and their primary components would aid in enhancing the readers' comprehension.

Thank you for your comment. We included more details about the Deurenberg and Hall TEI equation, and referred to the next subsections and S1 Appendix for more details.

***Figure 1*** ***summarizes*** *each step of the modeling process****. Briefly, we first estimated baseline fat-free mass (FFM) and fat mass (FM) for the children and adolescents based on body mass index, age, and sex, using*** *an equation developed and validated by Deurenberg, et al [27].* ***This equation estimates the body fat percentage for children and adolescents, derived from a multiple linear regression.*** ***Baseline FFM and FM were used to predict the total energy intake per day (TEI) depending on sex, age and BMI category using a non-linear function proposed by*** *Hall et al. [28].* ***This*** ***TEI function considered children’s growth and was derived from the sum of two components: energy balance (= energy intake - energy expenditure), and energy expenditure.*** *We estimated the effect of the law on TEI in Mexican children under three different scenarios: 1) main scenario, using Mexican data to inform the intervention effect, 2) sensitivity scenario varying law compliance, and 3) sensitivity scenario, using meta-analytic data to inform the effect of the intervention. Considering a business-as-usual scenario (BAU; no intervention) and the estimated intervention effect on TEI, we used Hall´s weight change microsimulation model to estimate the potential impact on body weight and obesity prevalence of children and adolescents that could be achieved one year after implementing the intervention in Mexican schools.* ***A more detailed description of the modeling process is presented in the next subsections and in S1 appendix.*** *Model inputs are presented in Table C in S1 Appendix.*

6. Method: the authors stated, "...without considering any previous trends or externalities that could influence changes in Total Energy Intake (TEI). As such, no increase in obesity prevalence is accounted for over one year of simulation. Furthermore, it is assumed that children's and adolescents' height will progress in line with the World Health Organization's height-for-age standards." Given the documented rise in childhood obesity in Mexico, the decision to exclude trend data from the simulation warrants scrutiny. It is essential to question whether there is empirical support for this key assumption, which significantly impacts the study's projections and findings.

Thank you for the comment. Adding an increasing obesity trend to the model would add another layer of complexity. We are considering growth to the model and increasing TEI and weight due to growth (Table I in S1 appendix). As we simulated the intervention over one year, the possible changes in obesity are likely to not affect the results much. Also in many countries, obesity increase is reducing or in plateau, so adding another source of uncertainty could be ambiguous. We commented this further in limitations, as follows:

***Our model considers a growth-related increase in TEI (Table I in S1 Appendix), but does not include an increase in TEI due to past obesity trends over time. However, this assumption should have little effect on estimates, given the one-year span of our simulation.***

Reviewer #3: Thank you for the opportunity to review this interesting paper. This research provides an opportunity to consider the potential impact of a national policy upon dietary intakes of children through a microsimulation study.

The researchers used appropriate methods to generate values for inclusion in the models to estimate the impact of a mandatory prohibition of sales of NEDFBs in schools upon NEDFB intakes and subsequent changes in BMI and obesity prevalence. The researchers considered multiple scenarios to provide a range of possible effect estimates, such as varying compliance. This is a very important consideration given the evidence of wide variation in implementation of such approaches, so provides more conservative estimates. It also considered potential compensatory consumption through other foods, again providing more conservative estimates.

A good level of detail is provided, particularly in the supplementary document. The paper is well written and draws appropriate conclusions, acknowledging some key limitations.

The paper may benefit from some additional detail or consideration of the following:

Introduction:

*	Additional text on the existing/planned monitoring / enforcement in place with regards to the 2010 guidelines to regulate the food environment of the National Education System.

Thank you for your comment. As you suggested, we include more detail about the new regulations to improve the monitoring and reinforcement of the food environment in the fourth paragraph of the introduction section as follows:

***In December 2023, the Mexican Senate approved a reform to strengthen the General Education Law [24]. This reform law implied a structural change in the regulatory framework, emphasizing direct government involvement in assuring compliance. The approved legislation mandates the Secretary of Education to issue new General Guidelines for the preparation, sale and distribution of prepared and processed foods and beverages in schools. Guidelines are under development, but the main changes to the law include the ban on advertising unhealthy food, expand the law to venues near schools, standardize the nutritional criteria to front of package labels proposed in the General Law of Health (Art 212) (supplementary table 2)[25], promote healthier food alternatives prioritizing plain water and stronger sanctions for non-compliance. Also, to enhance compliance, the Secretary of Education will now monitor and ensure the enforcement of the new guidelines through school directors and supervisors, replacing the previous role of parental committees.*** *It is expected the law reform will promote better compliance, in the US, policies restricting SSB consumption in schools had higher impact when mandatory and when an administrative agency was designated as responsible for its implementation [26]. We aimed to estimate the expected impact of banning sales of non-essential energy-dense food and beverages (NEDFBs) in schools on total caloric intake and obesity prevalence of school-aged children and adolescents one year after its implementation, considering varying degrees of compliance.*

*	A brief overview of the school food system in Mexico to provide a picture of any potential commercial influences that might influence compliance with the new restrictions on sales and advertising of unhealthy food inside schools and surroundings e.g. is school food prepared by in-house caterers, centrally provided (e.g. local or national catering provider) or by private companies?

Thank you for the comment. As you suggested, we identified the lack of regulation on the advertising and sale of junk food both inside and around schools as key factors for low compliance in the previous guidelines; still, monitoring will be one of the main challenges faced by new guidelines. We integrated more detail in paragraphs three and four of introduction, as follows:

Introduction, third paragraph:

*In 2010, Mexico implemented national nutritional guidelines* ***as a part of the Sectoral Health Program 2007-2012****[19] to regulate* ***the preparation, sale and distribution of foods and beverages that exceeded recommended nutritional standards in elementary and middle schools*** *[20]. By 2014,* ***through a reform of the General Education Law,*** ***these guidelines*** *became mandatory and punishable* *[21]****. The law provided an exception for Fridays, when sales of all products were allowed (supplementary table 1)****. T****his law was poorly implemented; between 2012 and 2015,*** *only 27% of food sold in schools met the nutritional criteria [22]. In 2018 in Northwest Mexico only 15% of schools had fully implemented the guidelines [23].* ***Low compliance has been explained by the poor dissemination and knowledge about the guidelines, lack of monitoring, enforcement and sanctions, failure to ban junk food advertising, and not applying restrictions to venues near schools*** *[22,23]****.*** *Valuable lessons were learned from this experience: 1) increase parental involvement, 2) offer healthy substitutes for popular foods and beverages, 3) engage food delivery staff and teachers, 4) provide free drinking water, and 5) regulate advertising of unhealthy food in and around schools [22,23].*

Introduction, fourth paragraph:

***In December 2023, the Mexican Senate approved a reform to strengthen the General Education Law*** *[24]****. This reform law implied a structural change in the regulatory framework, emphasizing direct government involvement in assuring compliance. The approved legislation mandates the Secretary of Education to issue new General Guidelines for the preparation, sale and distribution of prepared and processed foods and beverages in schools. Guidelines are under development, but the main changes to the law include the ban on advertising unhealthy food, expand the law to venues near schools, standardize the nutritional criteria to front of package labels proposed in the General Law of Health (Art 212) (supplementary table 2)*** *[25]****,*** *promote healthier food alternatives prioritizing plain water* ***and stronger sanctions for non-compliance.*** ***The Secretary of Education will now assume the responsibility of monitoring and ensuring the enforcement of the new guidelines through school directors and supervisors, replacing the previous role of parental committees.*** *It is expected the law reform will promote better compliance, in the US, policies restricting SSB consumption in schools had higher impact when mandatory and when an administrative agency was designated as responsible for its implementation [26]. We aimed to estimate the expected impact of banning sales of non-essential energy-dense food and beverages (NEDFBs) in schools on total caloric intake and obesity prevalence of school-aged children and adolescents one year after its implementation, considering varying degrees of compliance.*

Methods:

*	Figure 1 - the quality of this image is poor (i.e. the resolution). Would suggest the authors provide a higher resolution image.

Thank you for your comment. As you suggested, we improved the quality of the figure:

***Figure 1. Step-by-step modeling process, showing inputs and outputs of the model.*** *Changes in body weight and obesity prevalence are estimated by comparing the intervention scenario with BAU one year after the intervention.* ****TEI****(t_0_, t_365_ ) is a vector with the daily total energy intakes from t_0_ to t_365_ = (t_0_+365), considering sex, age, baseline (initial) BMI category, Fat Mass (FM) and Fat-Free Mass (FFM). FM y FFM were estimated using the body fat percentage equation (%BF) developed and validated by Deurenberg et al.: FM = %BF*BW=(1.51*BMI - 0.7*Age - 3.6*Sex + 1.4)*BW/100 and FFM = BW - FM, where BW denotes body weight and BMI represents body mass index. ** SES = Socioeconomic status.*

*	Although multiple scenarios have been explored, the authors have not considered potential compensatory intake of NEDFBs outside of school (i.e. if banned in school, would these be purchased/consumed at other points in the day instead) i.e. intake would not change over 24 hours, or the effect of the intervention upon 24 hour intake is diluted (or there may even be over-compensation of consumption of these foods/drinks outside of school). This is perhaps similar to scenario 1b but the compensatory effect may be different. Overcompensation outside of school (or even the potential for this policy to drive children to bring these foods/drinks into school from home, if they can no longer be purchased at school) could perhaps be a 'worse case scenario' or unintended harmful effect.

Thank you for your comment. We estimated compensation from Chile’s results, including compensation outside schools, after three interventions (warning labeling, banning sales at schools, and marketing restrictions). This compensation captures changes inside and outside school; we assumed similar reductions inside and outside and applied this proportion to calories eaten inside schools. The considered compensation was 81% for food which we think is already considerable. However, another study calculating compensation for specific nutrients, such as sugar found a 80% compensation for children and a complete compensation for adolescents. Also, the limitation is that we are not capturing compensation heterogeneity between children, adolescents, and adults. We modified the text in methods and added a line in the limitations as follows:

*Methods*

*We simulated our main scenario by assuming 100% compliance, i.e., that these proportions of the TEI would be eliminated if the law effectively banned NEDFBs. We tested this scenario under two different assumptions:*

*b. Energy compensation, in which a part of the calories reduced is compensated by consuming other food and beverages in schools. We estimated the expected percentage of caloric compensation using results from a previous study in Chile, which estimated the consumption of regulated and non-regulated food and beverages at the household level after implementing a package of interventions, including warning labels, banning sales of unhealthy food and beverages at schools, and marketing restrictions.[32] After implementation, calories from regulated food and beverages decreased 33.1 and 16.3 kcal/capita/day, respectively, while calories from non-regulated items increased 26.8 in food and 6.3 kcal/capita/day in beverages. With these data, we estimated a compensation rate on foods of 81% (=26.8/33.1), and 38.7% for beverages(=6.3/16.3).* ***The compensation rate considers compensation inside and outside schools, but this was applied to calories eaten inside schools and it fails to capture potential differences by age group.***

Limitations

*Some limitations must be acknowledged. First, the total impact of the intervention may be underestimated because we are using 24-h nutritional recall data from 2012 to estimate the calories from junk food consumed at school, which is known to underestimate unhealthy food consumption. Also, for children under 12 years of age, 24-h nutritional recall data obtains information from parents or guardians, who may not be well informed about their child’s consumption patterns at school. Still, ENSANUT is representative at the national level, allowing us to make population estimates generalizable to the Mexican context. Results may be overestimated because we are assuming that all children and adolescents go to school, but in the 2019-2020 cycle, 98.7% of 6–12-year-olds, 95.9% of 13–15-year-olds and 75.5% of 16-18-year-olds were enrolled in school[42]. Given the uncertainty of the compliance rate, we tested different compliance levels, but we assumed that it would be at least twice the compliance of the 2014 guidelines implemented in Mexico (15%)[23]. We are assuming that the banning effect and compliance rate are equal for public or private schools. Evidence exists that private schools had better compliance with the regulation than public schools[23], but the overall effect is likely to be small, as most Mexican children attend public schools[42]. Overestimation could also arise from* ***energy compensation, either from allowed food and beverages in school or through NEDFBs consumed outside of schools. We considered substantial levels of food compensation (81%) and beverages (39%), based on household-level estimates after the Chilean policies on food labeling, marketing, and sales in schools [32]. Another study from Chile after the same policies did not report caloric compensation, but compensation for specific nutrients. They reported an 80% sugar compensation for children, but complete sugar compensation for adolescents [17]. Our model considers a growth-related increase in TEI (Table I in S1 Appendix), but does not include an increase in TEI due to past obesity trends over time. However, this assumption should have little effect on estimates, given the one-year span of our simulation.*** *Finally, we are not considering the potential effect of banning junk food near schools, as our study only included the expected impact inside schools.*

Discussion:

*	It may be worth considering the potential equity impact of the policy - for example, might we see greater impact across socioeconomic strata or gender? (i.e. if NEDFBs make up a larger proportion of TEI for lower SES groups, the impact of the policy may be greater; likewise boys may typically have higher intakes.) This would be an interesting point in the discussion, given the global interest in health equity.

Thank you for your comment. Following your suggestion, we included a third sensitivity scenario considering the effect of the ban by socioeconomic status and sex. Description and results were added to the text as follows:

In the Methods section:

***4****.* ***Sensitivity scenario using Mexican dietary data to inform the intervention effect by socioeconomic status and sex.*** *For this scenario, the NEDFBs energy contribution was estimated by socioeconomic status (SES) and sex to assess the potential equity impact of the policy. As in the main scenario, we used the 24-hour dietary recall data from ENSANUT 2012 to estimate the energy contribution of NEDFBs to TEI in schools, and also considered both the energy compensation and no energy compensation assumptions. For females, NEDFBs intake in schools represented about 4.7%, 5.0%, and 6.5% of the TEI for the low, middle and high SES, respectively. For males, those values were about 2.3%, 3.5%, and 4.7% of the TEI for the low, middle and high SES, respectively.*

In the Results section:

***Figure 3 shows results stratified by sex and socioeconomic status. We found that the expected impact is estimated to be higher in females, mainly in higher SES, with an expected reduction of 1.1 kg/day/person considering compensation, which translates into 2.1 pp obesity reduction. Among males, we found a higher expected impact among high SES than low SES with a expected reduction of 0.9 kg/day/person considering compensation, which translates into 1.8 pp obesity reduction***

**Figure 3.** *Estimates of body weight, and obesity changes by socioeconomic status (SES) and sex one year after implementing the banning of non-essential, energy-dense food and beverages in schools in Mexico.*

*	Likewise, it would be good to consider whether we would expect to see differential compliance based on school characteristics? E.g. schools where the policy might have the greatest impact having low compliance.

Thank you for the comment. We could expect different compliance if the school is public or private. A previous study in schools in northwestern Mexico showed greater compliance in private versus public schools with 33.3 vs. 9.0% respectively. However, the majority of the schools in Mexico are public. Some other school characteristics could influence compliance, but we have no data on this. We stated the limitation in the manuscript as follows:

*Some limitations must be acknowledged. First, the total impact of the intervention may be underestimated because we are using 24-h nutritional recall data from 2012 to estimate the calories from junk food consumed at school, which is known to underestimate unhealthy food consumption. Also, for children under 12 years of age, 24-h nutritional recall data obtains information from parents or guardians, who may not be well informed about their child’s consumption patterns at school. Still, ENSANUT is representative at the national level, allowing us to make population estimates generalizable to the Mexican context. Results may be overestimated because we are assuming that all children and adolescents go to school, but in the 2019-2020 cycle, 98.7% of 6–12-year-olds, 95.9% of 13–15-year-olds and 75.5% of 16-18-year-olds were enrolled in school[42]. Given the uncertainty of the compliance rate, we tested different compliance levels, but we assumed that it would be at least twice the compliance of the 2014 guidelines implemented in Mexico (15%)[23]. We are assuming that the banning effect and compliance rate are equal for public or private schools.* ***Evidence exists that private schools had better compliance with the regulation than public schools[23]****, but the overall effect is likely to be small, as most Mexican children attend public schools[42]. Overestimation could also arise from* ***energy compensation, either from allowed food and beverages in school or through NEDFBs consumed outside of schools. We considered substantial levels of food compensation (81%) and beverages (39%), based on household-level estimates after the Chilean policies on food labeling, marketing, and sales in schools[32]. Another study from Chile after the same policies did not report caloric compensation, but compensation for specific nutrients. They reported an 80% sugar compensation for children, but complete sugar compensation for adolescents[17]. Our model considers a growth-related increase in TEI (Table I in S1 Appendix), but does not include an increase in TEI due to past obesity trends over time. However, this assumption should have little effect on estimates, given the one-year span of our simulation.*** *Finally, we are not considering the potential effect of banning junk food near schools, as our study only included the expected impact inside schools.*

*	The authors provided a critique of the dietary intake measure used in the limitations section. However, one additional point to explore would be how representative is the ENSANUT sample compared to the population (i.e. how generalisable is the ENSANUT to the study's population)?

Thank you for your comment. Due to the probabilistic design of the ENSANUT 2018 survey, it is representative at the national and at the state level for adults, adolescents, and children. Additionally, since it is a probabilistic survey, it is possible to quantify the uncertainty of its estimates using confidence intervals and make comparisons with other survey waves. We included the representativeness in the limitations, as follows:

*Some limitations must be acknowledged. First, the total impact of the intervention may be underestimated because we are using 24-h nutritional recall data from 2012 to estimate the calories from junk food consumed at school, which is known to underestimate unhealthy food consumption. Also, for children under 12 years of age, 24-h nutritional recall data obtains information from parents or guardians, who may not be well informed about their child’s consumption patterns at school.* ***Still, ENSANUT is representative at the national level, allowing us to make population estimates generalizable to the Mexican context.***

*	Although an overview of the meta-analysis used for the second sensitivity scenario (using meta-analytic data to inform the intervention effect) is provided, I would have liked to seen more on the meta-analysis to get a feel for whether the data that these values were based upon were from low/medium/high quality studies (e.g. if you only used high quality studies, what would be the expected intervention effect?)

Thank you for your comment, we acknowledge that the second sensitivity analysis is likely to overestimate the overall effect. We have added statements to both the methods and discussion sections to note that the studies used in the meta-analysis were mostly before-after and cross-sectional studies. Stronger study designs could better control for concurrent interventions and confounding, in smaller effects. For these reasons, we modeled scenarios of 25 and 50% of the meta-analysis effect. In practice, these types of studies are difficult to conduct, since randomized or staggered introduction of the law (e.g. by state) is rare, hampering an adequate prospective evaluation. We added more details from the meta-analysis and the relative reduction in TEI in section 4.3 the S1 Appendix.

*4.3 Sensitivity scenario using meta-analytic data to inform the intervention effect.*

*This scenario is based on the meta-analysis performed by Micha et. al, evaluating interventions in the school food-environment [17]. We reviewed* ***all*** *the studies included in Micha’s analysis and selected those that evaluated an intervention similar to ours* ***(n=6)****, [18] ie, interventions that banned certain types of food and beverages.* ***Since the meta-analysis included the period March 2014 to December 2017, we performed a rapid review with the same search terms as Micha, et al., (“Effectiveness of school food environment policies on children’s dietary behaviors: A systematic review and meta-analysis”) from December 2017 until 2022 and we identified*** *another study* ***published in 2020*** *estimating kcal reduction after modifying the nutrition standards and banning processed foods and beverages inside the school [19].*

*Using a random-effect model, we obtained a reduction in total energy intake of 177.0 kcal (95% CI [-241.9, -112.2]) (Pooled in Table H).* ***Given the heterogeneity of the total energy intakes, we estimated the relative reduction from the pooled TEI of the control groups (1,811.0 kcal)*** *–before the intervention or unexposed to the intervention.* ***This reduction represented a change of -9.8% (95%CI [-13.4%, -6.2%]) from TEI. The studies were observational without an ideal control group, so*** *there is likely an overestimation of the effect,* ***especially when compared to Ensanut’s estimates****.* ***Hence,*** *we consider three effects: 100% (point estimate), 50%, and 25% of the effect.*

Best wishes, Dr Marie Murphy
