# Supplementary material for: Predicted impact of banning nonessential, energy-dense food and beverages in schools in Mexico: A microsimulation study
Source: PLoS Med. 2024 May 10;21(5):e1004394. doi: 10.1371/journal.pmed.1004394 (PMC11086919; doi:10.1371/journal.pmed.1004394)
Supplement: Attachment — Submitted filename: Comments banning R2.docx [file pmed.1004394.s003.docx]

Dear Dr. Barrientos-Gutierrez,

Thank you very much for re-submitting your manuscript "Predicted impact of banning non-essential, energy-dense food and beverages in schools in Mexico: a microsimulation study" (PMEDICINE-D-23-03317R2) for review by PLOS Medicine.

I have discussed the paper with my colleagues and the academic editor and it was also seen again by the reviewers. I am pleased to say that provided the remaining editorial and production issues are dealt with we are planning to accept the paper for publication in the journal.

Given the minor nature of the remaining comments (see comment from reviewer #3), we expect to receive your revised manuscript within the next few days week (Friday, 22nd of March, 2024). Please email me (kjanin@plos.org) if you have any questions or concerns.

The remaining issues that need to be addressed are listed at the end of this email. Any accompanying reviewer attachments can be seen via the link below. Please take these into account before resubmitting your manuscript:

***Please note while forming your response, if your article is accepted, you may have the opportunity to make the peer review history publicly available. The record will include editor decision letters (with reviews) and your responses to reviewer comments. If eligible, we will contact you to opt in or out.***

In revising the manuscript for further consideration here, please ensure you address the specific points made by each reviewer and the editors. In your rebuttal letter you should indicate your response to the reviewers' and editors' comments and the changes you have made in the manuscript. Please submit a clean version of the paper as the main article file. A version with changes marked must also be uploaded as a marked up manuscript file.

Please also check the guidelines for revised papers at <http://journals.plos.org/plosmedicine/s/revising-your-manuscript> for any that apply to your paper. If you haven't already, we ask that you provide a short, non-technical Author Summary of your research to make findings accessible to a wide audience that includes both scientists and non-scientists. The Author Summary should immediately follow the Abstract in your revised manuscript. This text is subject to editorial change and should be distinct from the scientific abstract.

We ask every co-author listed on the manuscript to fill in a contributing author statement. If any of the co-authors have not filled in the statement, we will remind them to do so when the paper is revised. If all statements are not completed in a timely fashion this could hold up the re-review process. Should there be a problem getting one of your co-authors to fill in a statement we will be in contact. YOU MUST NOT ADD OR REMOVE AUTHORS UNLESS YOU HAVE ALERTED THE EDITOR HANDLING THE MANUSCRIPT TO THE CHANGE AND THEY SPECIFICALLY HAVE AGREED TO IT.

Please ensure that the paper adheres to the PLOS Data Availability Policy (see <http://journals.plos.org/plosmedicine/s/data-availability>), which requires that all data underlying the study's findings be provided in a repository or as Supporting Information. For data residing with a third party, authors are required to provide instructions with contact information for obtaining the data. PLOS journals do not allow statements supported by "data not shown" or "unpublished results." For such statements, authors must provide supporting data or cite public sources that include it.

Please review your reference list to ensure that it is complete and correct. If you have cited papers that have been retracted, please include the rationale for doing so in the manuscript text, or remove these references and replace them with relevant current references. Any changes to the reference list should be mentioned in the rebuttal letter that accompanies your revised manuscript.

Please note, when your manuscript is accepted, an uncorrected proof of your manuscript will be published online ahead of the final version, unless you've already opted out via the online submission form. If, for any reason, you do not want an earlier version of your manuscript published online or are unsure if you have already indicated as such, please let the journal staff know immediately at plosmedicine@plos.org.

If you have any questions in the meantime, please contact me (kjanin@plos.org) or the journal staff on plosmedicine@plos.org.

We look forward to receiving the revised manuscript by Mar 22 2024 11:59PM.

Sincerely,

Katrien Janin, PhD

Senior Editor

PLOS Medicine

[plosmedicine.org](http://plosmedicine.org/)

------------------------------------------------------------

Note from the editors:

We like to thank the authors for addressing fully our comments, and for the the additional clarity you have provided provided in the manuscript. (but please do see the outstanding comment from reviewer #3)

As a general comment, please note that supplementary materials are not checked and will be posted as supplied by the authors. Therefore, please double check and ensure you appropriate cite the Supplementary Information in your manuscript. Please cite your Supporting Information as outlined here: <https://journals.plos.org/plosmedicine/s/supporting-information> - Please note you may use almost any description as the item name of your supporting information as long as it contains an "S" and number. For example, “S1 Appendix” and “S2 Appendix,” “S1 Table” and “S2 Table.

We double-checked the supplementary materials and changed the name of tables to include S1, S2 Tables as suggested; calls to these tables were also changed in the main text.

I look forward to receiving the revised version of your manuscript, and issuing the editorial accept for you paper,

Best wishes,

Katrien Janin

(kjanin@plos.org)

------------------------------------------------------------

Comments from Reviewers:

Reviewer #2: In my opinion, the authors have adequately addressed my and other reviewers' comments and concerns. Congratulations!

Reviewer #3: Dear authors,

Thank you for taking the time to address my comments. Your responses are well considered, and I thank you for the additional clarity provided in the manuscript to address these points.

I would like to ask for one further clarification please, regarding "A brief overview of the school food system in Mexico to provide a picture of any potential commercial influences that might influence compliance with the new restrictions on sales and advertising of unhealthy food inside schools and surroundings e.g. is school food prepared by in-house caterers, centrally provided (e.g. local or national catering provider) or by private companies?" I am still not clear on the school food system in Mexico and potential additional motives for low compliance - for example, is the food sold in schools done so through private contracts with catering companies (in which case there may be a strong profit motive for non-compliance via increased sales) or is food sold via local/national/school catering services (i.e. not-for-profit)? This may help to provide some context around Mexico's school food system for those unfamiliar with it.

R. Thank you for your excellent comment. We added the following in the introduction and the discussion.

Introduction

Mexico does not have a formal school meal program that covers all schools, except for a school breakfast program funded by the government that operates in less than 50% of elementary schools. Instead, most schools have school cafeterias that are operated privately (the appointment and training of the provider is the responsibility of the Secretary of Education). In 2010, Mexico implemented national guidelines as a part of the Sectoral Health Program 2007-2012[19] to regulate the preparation, sales, and distribution of foods and beverages that exceeded recommended nutritional standards in elementary and middle schools [20]. By 2014, through a reform of the General Education Law these guidelines became mandatory and punishable (S1 Table presents a list of prohibited food and beverages) [21]. The law provided an exception for Fridays, when all products were allowed. This law was poorly implemented: between 2012 and 2015, only 27% of food sold in schools met the nutritional criteria [22] and in 2018 in Northwest Mexico only 15% of schools had fully implemented the guidelines [23]. Low compliance has been explained by the poor dissemination and knowledge about the guidelines, lack of monitoring, enforcement and sanctions, failure to ban junk food advertising, and not applying restrictions to venues near schools [22,23]. Valuable lessons were learned from this experience: 1) increase parental involvement, 2) offer healthy substitutes for popular foods and beverages, 3) engage food delivery staff and teachers, 4) provide free drinking water, and 5) regulate advertising of unhealthy food in and around schools [22,23].

Discussion

The school context is very important for implementing and impacting health interventions. Our sensitivity analysis, using a meta-analytical input, showed a higher expected effect of the intervention. However, studies included in the meta-analysis were conducted in high-income countries, where the infrastructure and organization of school food services is better and where compliance could also be higher than in Mexico. Previous experience with the guidelines issued in 2010 showed that the lack of dissemination and training, the lack of regulations regarding the food sold near schools (e.g. by ambulatory food vendors), and the lack of monitoring were key elements in the schools’ low compliance [22,23]. Furthermore, school cafeterias share a small percentage of profits with schools, which are used to buy essential supplies, improve facilities, or finance school activities; thus, food sales are an economic incentive for schools. With the 2023 modification to the law, some of these limitations are addressed, including the promotion of endemic and natural foods from each region to reduce the incentive to sell processed foods, as well as a mechanism for implementation, evaluation, monitoring and sanctions for non-compliance. However, considering that the degree of compliance is still uncertain and may vary for different reasons, including possible economic incentives for schools to sell processed foods, we evaluated its impact in our sensitivity analysis, showing that if all schools comply with the law, the intervention could reduce 0.8 kg/person, but if only 30% of schools comply with it, the expected reduction could be 0.2 kg/person.

Unfortunately I am still finding the figure 1 to be of low resolution - but perhaps this can be fixed at the editorial stage.

Thanks, we changed all figures to pdf to improve resolution.

I am satisfied that my other comments have been addressed satisfactorily.

Best wishes, Dr Marie Murphy

1.	[Global Survey of School Meal Program. *GCNF*](http://paperpile.com/b/e4BO5q/qJQU) <https://gcnf.org/country-reports/> [(2022).](http://paperpile.com/b/e4BO5q/qJQU)

2.	[Hugues, Y., Díaz-Zavala, R. G., Quizán-Plata, T., Corvalán, C. & Haby, M. M. Poor compliance with school food environment guidelines in elementary schools in Northwest Mexico: A cross-sectional study. *PLoS One* **16**, e0259720 (2021).](http://paperpile.com/b/e4BO5q/Jpv7)
